# Tor forms a dimer through an N-terminal helical solenoid with a complex topology

**DOI:** 10.1038/ncomms11016

**Published:** 2016-04-13

**Authors:** Domagoj Baretić, Alex Berndt, Yohei Ohashi, Christopher M. Johnson, Roger L. Williams

**Affiliations:** 1 MRC Laboratory of Molecular Biology, Cambridge CB2 0QH, UK

## Abstract

The target of rapamycin (Tor) is a Ser/Thr protein kinase that regulates a range of anabolic and catabolic processes. Tor is present in two complexes, TORC1 and TORC2, in which the Tor–Lst8 heterodimer forms a common sub-complex. We have determined the cryo-electron microscopy (EM) structure of Tor bound to Lst8. Two Tor–Lst8 heterodimers assemble further into a dyad-symmetry dimer mediated by Tor–Tor interactions. The first 1,300 residues of Tor form a HEAT repeat-containing *α*-solenoid with four distinct segments: a highly curved 800-residue N-terminal 'spiral', followed by a 400-residue low-curvature 'bridge' and an extended ‘railing' running along the bridge leading to the 'cap' that links to FAT region. This complex topology was verified by domain insertions and offers a new interpretation of the mTORC1 structure. The spiral of one TOR interacts with the bridge of another, which together form a joint platform for the Regulatory Associated Protein of TOR (RAPTOR) regulatory subunit.

## Abstract (web summary)

The target of rapamycin (Tor) is a Ser/Thr protein kinase that regulates a wide range of anabolic and catabolic processes. Here the authors describe a sub-nanometer cryo-EM structure of a yeast Tor–Lst8 complex and propose an overall topology that differs from that previously suggested for mTORC1.

## Graphical abstract

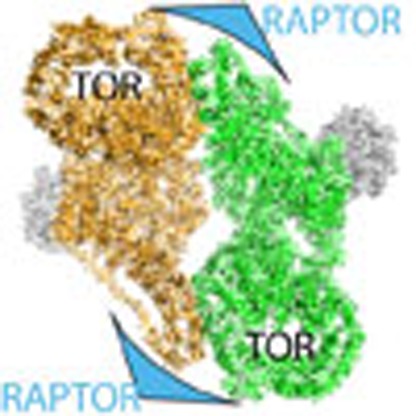

The Ser/Thr protein kinase Tor is a hub for cellular homeostatic regulation^1^,^2^,^3^,^4^. While some yeast species such as *Saccharomyces cerevisiae* have two genes that encode Tor1 and Tor2 proteins^5^,^6^,^7^,^8^, mammalian cells have a single gene encoding mTOR^9^,^10^. Both Tor and mTOR occur in two complexes known as TORC1 and TORC2 (refs 11, 12, 13, 14, 15). TORC1 controls cell size/volume by regulating protein translation, ribosome biogenesis and autophagy^2^,^16^,^17^. The TORC1 complex is activated by amino acids^18^ and in mammalian cells also by growth factors, insulin, energy levels and oxidative stress^2^. TORC2 regulates lipid metabolism, actin cytoskeleton and the survival of DNA damage^4^,^16^,^19^,^20^. The TORC1 complex consists of three core subunits: the catalytic subunit, Tor1 or Tor2 in yeast (mTOR in mammals), Lst8 (mLST8) and Kog1 (RAPTOR in mammals)^11^,^14^,^15^,^21^,^22^. The core subunits of the TORC2 complex are Tor2 (mTOR in mammals), Lst8 (mLST8 in mammals), Avo1 (mSin1 in mammals) and Avo3 (RICTOR in mammals)^12^,^13^,^21^,^23^,^24^. Lst8 is a requisite component for both TORC1 and TORC2 (refs 15, 22, 25). The activity of TORC1 is regulated by direct interaction with small GTPases, such as Gtr1/Gtr2 (Rag heterodimers in mammals) that regulate Tor localization and activity^26^,^27^,^28^,^29^,^30^.

The prominent role of mTORC1 in the PI3K pathway has led to widespread efforts to develop mTOR inhibitors that could act as anti-cancer therapeutics^31^. The best-characterized substrates of mTORC1 are eIF4E-binding protein (4E-BP1) and ribosomal S6 kinase (S6K)^32^. Through these substrates, mTORC1 controls cap-dependent translation initiation and elongation^33^. Tor signalling motifs are present in these substrates and are recognized by the RAPTOR subunit^34^.

mTOR is a member of the phosphoinositide 3-kinase related kinases (PIKKs), which also include Ataxia telangiectasia mutated (ATM), ATR (ATM related), Suppressor of morphogenesis in genitalia-1 (SMG-1), Transformation/Transcription Domain-Associated Protein (TRRAP) and DNA-dependent protein kinase, catalytic subunit (DNA-PKcs)^35^. PIKKs share a catalytic core consisting of a kinase domain flanked by a helical FAT domain (named after FKBP12 Rapamycin Associated Protein (FRAP)–ATM–TRRAP) at the N-terminus and a short FAT-C domain at its C-terminus^36^. We will refer to this catalytic core as the FAT plus KINase domain (FATKIN). The recent crystal structure of human mTOR FATKIN in a complex with the small WD40-repeat protein mLST8 (PDB ID 4JSV) provided a wealth of information regarding substrate access to the kinase domain and the catalytic mechanism of the enzyme^37^. The FAT region consists of four domains, three tetratrico peptide repeat domains (TRD1, TRD2 and TRD3) and a HEAT-repeat domain (HRD). The mTOR kinase domain has a 100-residue insertion in its N-lobe known as the FRB (FKBP12-rapamycin binding) domain that is unique to a subset of the PIKKs, including SMG-1, DNA-PKcs and TRRAP^37^. The FRB helps recruit substrates, restricts substrate access into the active site and is an essential component of the mechanism whereby the fungal inhibitor rapamycin (sirolimus) inhibits mTOR activity^37^,^38^.

The FATKIN represents no more than half of the protein for any of the PIKKs. All PIKKs have vast N-terminal regions that have been proposed to consist of helical solenoids formed from HEAT repeats^39^, however, only limited structural information is available for this portion of the PIKKs. The N-terminal solenoids of the PIKKs are important for the interactions that regulate and adapt the activity of the enzyme complexes. The PIKK SMG-1 that regulates nonsense-mediated mRNA decay interacts with its co-factors SMG-8 and SMG-9 through its N-terminal HEAT repeats^40^. The largest HEAT-repeat region among the PIKKs is the 3,000 residue N-terminal region of DNA-PK^41^. The DNA-PK HEAT region binds KU proteins and forms a wide circular structure encompassing what is thought to be the DNA-binding domain of the enzyme^41^. A deletion of the HEAT repeats in yeast Tor2 delocalized the protein from the plasma membrane^42^. A *tor1* mutant in which a 3xGFP tag was inserted, after residue Asp67 was only partially functional^43^. The N-terminal HEAT repeats of mTOR bind RAPTOR^14^. In mTOR, ATM and ATR, the N-terminal portion of *α*-solenoid contains the Tel2 binding site. Tel2 is a component of a chaperone supercomplex Hsp90-TTT-R2TP that regulates the stability of the PIKKs^44^,^45^,^46^.

Despite the importance of the N-terminal *α*-solenoid for stability and function of the PIKKs, only low-resolution structures have been reported for full-length Tor and mTOR: a 25 Å resolution negative-stain EM structure of the *S. cerevisiae* Tor1 (EMDB-1360) and a Tor1/Kog1 complex (EMDB-1361)^47^ as well as a 26 Å resolution structure of the human mTORC1 (mTOR–mLST8–RAPTOR–PRAS40) complex (EMDB-5197)^48^. However, because of the low resolution, they provide limited information regarding the organization of the enzyme. Focusing on the heterodimer that forms the core of both TORC1 and TORC2, we have determined a 6 Å resolution structure of the full-length Tor–Lst8 complex from the thermotolerant yeast *Kluyveromyces marxianus* (KmTor). Our structure shows that KmTor–Lst8 forms a dimer of heterodimers, in which the two KmTor subunits embrace each other with their helical solenoids. The dimeric interface is distinct from the dimer interface previously described for the low-resolution structure of human mTORC1 (EMDB-5197)^48^, and we suggest a plausible reinterpretation of this structure. During revision of the current manuscript, a cryo-EM structure of mTORC1 was reported^49^. While the density of mTORC1 is similar to the density that we see for KmTor–Lst8, the topology that we have experimentally verified, using a series of landmarks in the structure fills in gaps in understanding the mTORC1 structure.

## Results

### Overall cryo-EM structure of KmTor–Lst8

**Figure 1:**
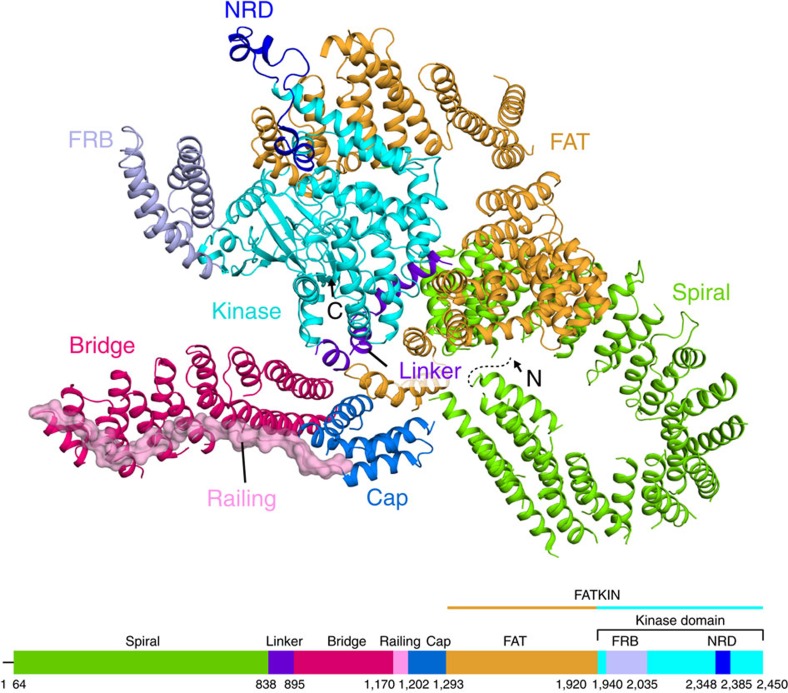
Fold of the KmTor monomer. The ribbon diagram of the KmTor monomer is illustrated above a bar showing the domain organisation of KmTor. Domain coloring of the bar matches the coloring of the ribbons. The dashed line (on the model) and a straight line (at the beginning of the bar showing the domain organization) indicate a portion of the KmTor N-terminus that is not present in the cryo-EM map.

Our cryo-EM structure of the KmTor–Lst8 complex is a dimer of heterodimers with two-fold rotational (C2) symmetry. The structure of each monomer shows an extensive N-terminal helical solenoid with a complex topology that precedes the FATKIN (Fig. 1). The Fourier shell correlation plot suggests that the overall resolution of the structure is ∼6 Å (Supplementary Fig. 1a), with the highest local resolution (5 Å) corresponding to the FATKIN and the N- and C-terminal regions of the *α*-solenoid that contact the FATKIN (Supplementary Fig. 1b). The lowest resolution region (7–8 Å) corresponds to the middle part of the spiral in the N-terminal helical solenoid. A plot of the orientations of the particles used for the structure determination shows two peaks, a top view and a side view, however, there is a broad distribution over all orientations, including the key equatorial views, to provide sufficient structural information for a reliable model (Supplementary Fig. 2). The KmTor–Lst8 complex used for cryo-EM was purified by affinity and ion-exchange chromatography (Supplementary Fig. 3a,b). It forms a stable assembly of about 600 kDa, consistent with a predominant dimer of 1:1 heterodimers, as determined by multi-angle light scattering (MALS) (Supplementary Fig. 3c), and it is catalytically active (Supplementary Fig. 3d).

### Structure of the FATKIN

**Figure 2:**
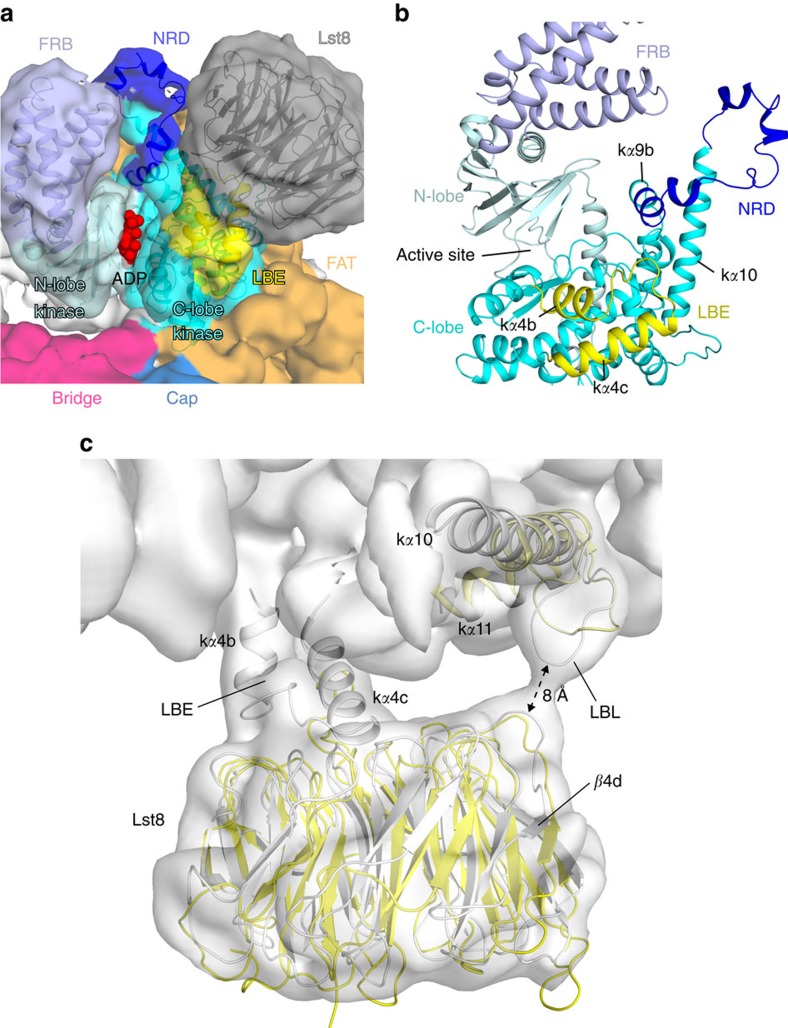
Negative regulatory domain (NRD) of KmTor. (**a**) The model of the KmTor active site with the superimposed KmTor–Lst8 cryo-EM density. Domain coloring of the density matches coloring of the ribbons (only KmTor kinase domain and KmLst8 are shown). The active site is marked by an ADP (red) that was modelled using the human mTOR FATKIN crystal structure (PDB ID 4JSV)^37^. (**b**) A close-up view of the NRD (dark blue) between helices k*α*9b and k*α*10. The NRD, the Lst8-binding element (LBE, yellow) and the FRB restrict access to the active site. (**c**) View of the KmTor–Lst8 interface in the model (grey) with the KmTor–Lst8 cryo-EM density superimposed (for display, densities in the map were normalised by subtracting the mean density in the map and dividing by the standard deviation of the densities. The map shown was contoured at 6 times the standard deviation, 6*σ*). The Lst8-binding element (LBE) and the Lst8-binding loop (LBL) of KmTor form interactions with the Lst8. For comparison, the same elements from human mTOR–mLST8 (PDB ID 4JSV, yellow)^37^ are superimposed on the KmTor–Lst8.

The human mTOR FATKIN (PDB 4JSV)^37^ fits readily in the KmTor–Lst8 density and occupies about half of the density for each monomer (Supplementary Fig. 4). The models for the Tor subunits in the dimer each have a FATKIN that agrees closely (RMSD 2.9 Å for 1014 residues, TM score^50^ of 0.9) with the structure of this fragment of the human mTOR that was reported previously (Supplementary Fig. 5)^37^. Consequently, we have kept the names of the secondary structure elements in the FATKIN as originally described (Supplementary Figs 5 and 6). The kinase domain has the two-lobed architecture common to the PI3Ks, with the ATP-binding site between the N-lobe and the C-lobe (Fig. 2a, Supplementary Fig. 7). In contrast to the PI3Ks, however, Tor has a domain known as the FRB domain (helices f*α*1–f*α*4, Supplementary Figs 6 and 7), which forms a prominent insertion in the N-lobe of the kinase domain. Additionally, the Lst8-binding element (LBE) forms an insertion (helices k*α*4b and k*α*4c) in the C-lobe. As in the human mTOR kinase domain structure, the FRB domain and the LBE of KmTor are arranged on either side of the catalytic cleft (Fig. 2). However, in KmTor, there is an additional element of structure protruding between the LBE and the FRB that further restricts entry to the active site (Fig. 2a,b). This feature includes the k*α*9b insertion that is unique with respect to PI3Ks (residues 2425–2436 in human mTOR and 2343–2352 in KmTor), which is part of a larger region that has been referred to as the negative regulatory domain (NRD) in mTOR (residues 2430–2492 in human mTOR and 2348–2385 in KmTor), since its deletion leads to an increase in activity^51^,^52^. In the human mTOR FATKIN structure (PDB ID 4JSV), this region is disordered (residues 2437–2491). For the KmTor structure, we observe density for the NRD, although somewhat weaker than the average density in the model (Fig. 2a,b). The NRD forms a bulbous protrusion in the kinase C-lobe leaving little space between the FRB and Lst8 to accommodate substrate entry into the deep active-site cleft (Fig. 2a,b). We have built a plausible NRD model, however, at this resolution, some uncertainty remains.

### Interaction of KmTor with KmLst8

The LBE in the C-lobe of the kinase domain of KmTor forms the most extensive interaction with KmLst8, similarly as in human mTOR–mLST8 (ref. 37). In the KmTor–Lst8 complex, the Lst8 subunit is slightly shifted relative to what was observed for the crystal structure of the human mTOR FATKIN–mLST8 complex (Fig. 2c). In the human mTOR–mLST8 structure, the mLST8 is involved in a contact with the HRD domain of another molecule in the crystal (PDB ID 4JSV^37^), and the lack of a crystal-packing constraint in KmTor–Lst8 might have resulted in the Lst8 shift that we see. Because of the shift in the orientation of Lst8 in the KmTor–Lst8 complex cryo-EM structure, there appears to be a direct interaction between the KmLst8 and a loop between k*α*10 and k*α*11 of the kinase domain (residues 2411–2419, corresponding to human mTOR 2510–2516) that we refer to as the Lst8-binding loop, LBL (Fig. 2c, Supplementary Fig. 6).

### Structure of the N-terminal *α*-solenoid

The N-terminal *α*-solenoid cradling the FATKIN consists of four distinct regions. The N-terminus of KmTor forms a highly curved spiral consisting of about 800 residues arranged into 36 helices (residues 64–837). This spiral has an elliptical projection with dimensions of about 70 Å × 83 Å (distances between outer rims, Fig. 1, Supplementary Fig. 8). Because side-chain density is not apparent at this resolution, it was not possible to establish an unambiguous register of the sequence with the density. To reduce uncertainty, we established a prominent landmark in the N-terminal spiral, by creating a variant of KmTor with tandem Red Fluorescent Proteins (RFP) inserted between residues 323 and 324. The position of the tandem RFP density in the structure allowed us to establish the direction of the spiral (Supplementary Fig. 9a,b,c). The N-terminus of the spiral is exposed, whereas the C-terminal end is against TRD2 of the FATKIN (Supplementary Fig. 10). The middle of the spiral shows considerable flexibility as suggested by the lower resolution of this region (Supplementary Fig. 1b). The spiral is followed by a linker (residues 838–894) that runs along the surface of the FATKIN to connect the spiral to a region that we will refer to as the 'bridge' (residues 895–1169, Fig. 1, Supplementary Fig. 10). The bridge, which has a much lower curvature than the spiral, consists of about 400 residues arranged in 14 helices (Supplementary Fig. 11). The spiral and the bridge consist entirely of helices with HEAT topology (helices N*α*1–N*α*51, excluding linker helices N*α*37a,b,c). The number and locations of helices in the N-terminal solenoid agree with a prediction based on an HHPRED analysis^53^,^54^,^55^.

The bridge is followed by a 32-residue linker that we refer to as the ‘railing' (residues 1170–1201) that folds back and runs over the entire length of the bridge, ending in several helices (N*α*52–N*α*56) that form a 'cap' (residues 1202–1292, Supplementary Fig. 11), which packs between the N-terminal end of the bridge and the TRD1 of the FATKIN (Fig. 1, Supplementary Figs 11 and 12). Because of the complex topology of the bridge and railing and in order to increase confidence in the directions of the railing and the bridge, we determined the cryo-EM structures of two additional constructs: one with a tandem RFP inserted at the beginning the railing (KmTor-1175RFP) and one with the RFP in the middle of the railing (KmTor-1190RFP). Consistent with our proposed topology, the cryo-EM density of the RFP for KmTor-1175RFP is connected to the beginning of the railing, farthest from the FATKIN (Supplementary Fig. 9d,e,f). The KmTor-1190RFP construct generates RFP density next to the concave side of the bridge where we would expect it from the model (Supplementary Fig. 9g,h,i).

The railing leads into the cap consisting of five helices. One connection between helices in the cap is not visible in the cryo-EM map, leaving uncertainty as to what the path of the polypeptide in the cap is. Nevertheless, the topology of the cap is not characteristic of a helical solenoid. The cap continues into TRD1 of the FATKIN (Supplementary Fig. 12), and the structure shows that TRD1 has five helices (*α*0, *α*1a, *α*1b, *α*2 and *α*3). Helix *α*0 and *α*1a are additional helices that were not present in the human FATKIN crystal structure^37^ and *α*1b formed part of a longer helix *α*1 in the human FATKIN (Supplementary Figs 5a,b and 6).

The element that has the largest deviation in KmTor from the crystallized human mTOR FATKIN^37^ is the helical hairpin at the end of the TRD2 domain in the FAT region (helices *α*11—*α*12 comprising residues 1495–1545 in KmTor, residues 1558–1606 in mTOR, Supplementary Fig. 5a,c)^37^. In the mTOR structure, this hairpin bends inward to establish a very limited intramolecular contact with a loop near the end of TRD1. In contrast, the *α*11–*α*12 hairpin of the full-length KmTor has an extensive intramolecular interface with the C-terminus of the spiral (helix N*α*36) (Supplementary Fig. 10).

### Tor–Tor dimer interface

**Figure 3:**
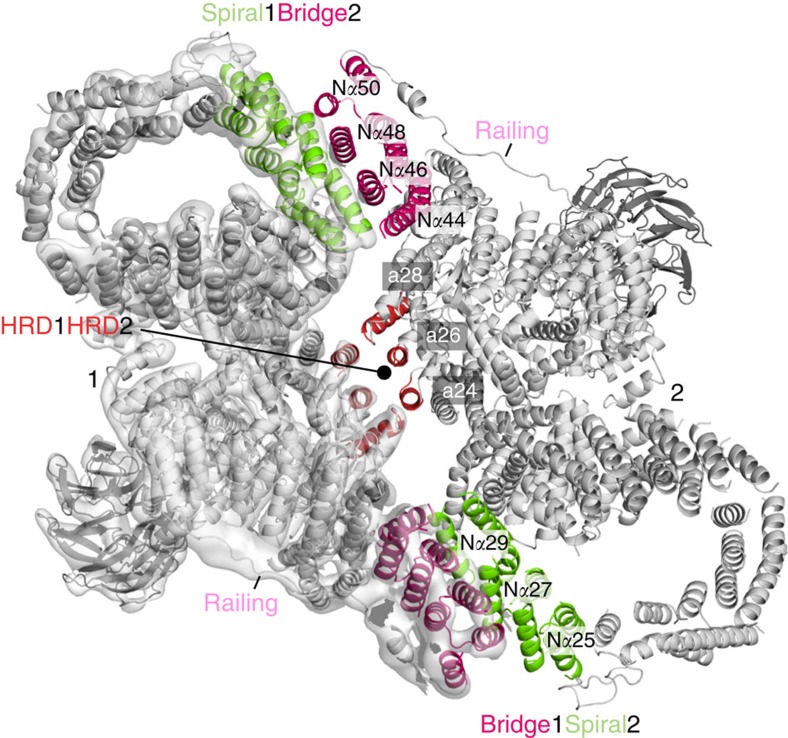
The interface between the two KmTor–Lst8 heterodimers in the assembly. The KmTor dimer assembly is mediated by interaction between HEAT-repeats. The primary contacts form between the helices in the spiral (N*α*25, N*α*27 and N*α*29) of one KmTor with helices in the bridge (N*α*44, N*α*46, N*α*48 and N*α*50) of the other. This interaction leads to a KmTor dimer that has two-fold rotational symmetry with the dyad axis between the two HRD domains (*α*24, *α*26 and *α*28) of the FATKINs where secondary contacts form. The cryo-EM density for one KmTor–Lst8 heterodimer is shown, contoured at 9*σ* everywhere except for the railing density, which is contoured at 4*σ*.

**Figure 4:**
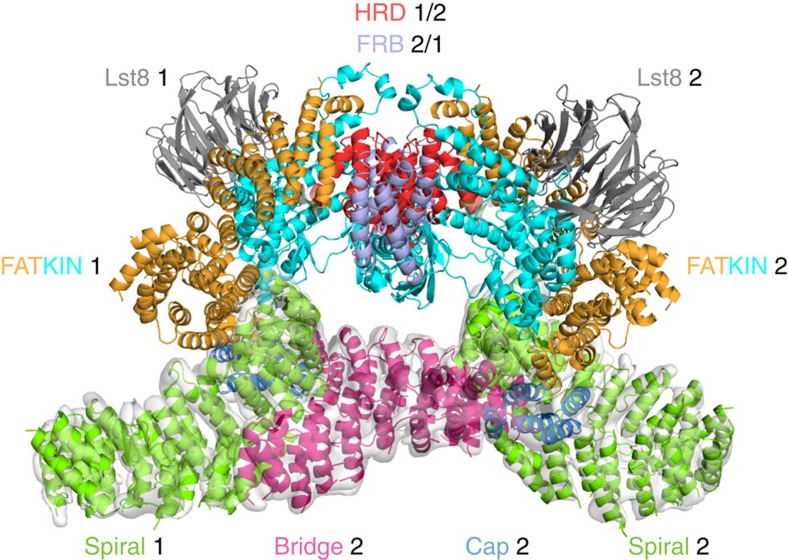
The FATKINs rest on a cradle made up of the N-terminal solenoids. The spiral (green) and bridge (magenta) domains associate with each other in the dimer of KmTor–Lst8 heterodimers to form a cradle on which the FATKINs rest. KmTor–Lst8 cryo-EM density is shown for the N-terminal solenoid region that precedes the FATKIN. The HRD that forms the interface between the FATKINs in the dimer is colored red and the FRB is colored light purple. The cryo-EM density was contoured as in Fig. 3.

The largest interface between the two KmTor subunits occurs at the periphery of the dimer and involves a region of the spiral (helices N*α*25, N*α*27 and N*α*29) of one molecule contacting the bridge (helices N\α44, N\α46, N\α48 and N\α50) of the other molecule (Fig. 3). The second, smaller interface consists of the HRD domain of one Tor interacting with the HRD of the other, via three helices (*α*24, *α*26 and *α*28) making intermolecular contacts at the center of the dimer (Fig. 3). This dimeric arrangement of KmTor places the two active sites facing away from each other, with the two FATKINs resting on a cradle made up of two N-terminal helical solenoids (Fig. 4).

## Discussion

The cryo-EM structure of KmTor–Lst8 reveals a dyad-symmetry dimer of heterodimers. The dimeric cryo-EM density that we observe for KmTor–Lst8 agrees closely with the density for the mTOR–LST8 component of a dimeric mTORC1 complex reported while this manuscript was under review^49^, suggesting that the presence of RAPTOR in mTORC1 has little impact on the structure of the Tor dimer (Supplementary Fig. 13a). Our structure of Tor indicates that the topology of the N-terminal solenoid that makes up about half of the protein is fairly complex and it helps resolve ambiguities present in the recent structure reported for mTORC1 (Supplementary Fig. 13b,c). Both KmTor and mTOR structures show prominent spiral and bridge regions (the spiral was referred to as the ‘horn' by Aylett *et al*.^49^), however, the proposed topologies differ in the directions of both the spiral and the bridge (Supplementary Figs 13 and 14). Only ‘direction 1' (Supplementary Fig. 13b), with the N-terminus of the spiral free and the C-terminus of the bridge farthest from the FATKIN, is consistent with the presence of the RFP landmarks that we have inserted into the spiral and the railing. Both the linker connecting the spiral to the bridge and the railing connecting the bridge to the cap, which are key to this topology, have weaker densities our cryo-EM maps of KmTor-Lst8 and KmTor–RFP-Lst8 suggesting some flexibility in these elements. One consequence of the ‘direction 1' topology is that the contact between the spiral and the bridge is intermolecular (Supplementary Fig. 13b) rather than the intramolecular contact that would be implied by ‘direction 2' (Supplementary Fig. 13c).

The KmTor–Lst8 dimer of heterodimers also can be readily fit into the EM density reported earlier for human mTORC1 (ref. 48) and very recently for yeast TORC2 (ref. 56), and the dimer relationship appears to be identical (Supplementary Fig. 15a,b).

**Figure 5:**
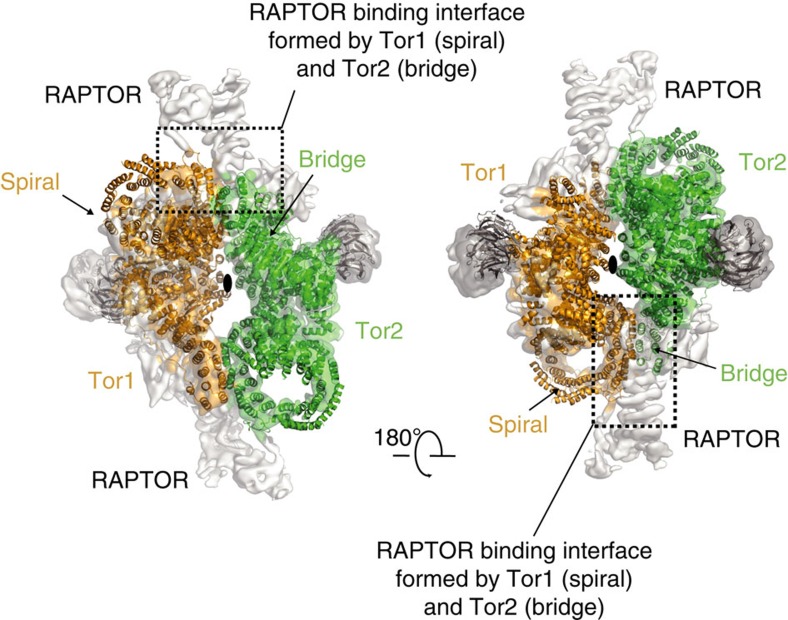
RAPTOR in the mTORC1 complex engages both mTOR molecules. Each of the two KmTor–Lst8 heterodimers are uniformly colored and shown as ribbons superimposed on the cryo-EM density for mTORC1 (EMDB-3212). Our topology of Tor implies that the RAPTOR in the mTORC1 complex engages both mTOR molecules through interaction with the spiral of one mTOR and the bridge of the dimer-related mTOR (interface enclosed in the dotted square).

The Kog1 (RAPTOR) subunit is important for interacting with substrates. Our KmTor–Lst8 structure provides some insight into the architecture of the RAPTOR-binding region. It has been reported that RAPTOR stabilizes the mTORC1 complex^48^. However, the recent cryo-EM study of the mTORC1 complex suggested that RAPTOR does not directly participate in the dimer formation^49^. In the mTORC1 complex, RAPTOR was proposed to associate with the bridge and the spiral (horn) of the same mTOR molecule and that this association indirectly stabilizes the mTOR dimer^49^. The alternative topology for mTOR suggested by our RFP landmarks implies that RAPTOR simultaneously binds to the spiral (horn) of one mTOR and the bridge of the dimer-related mTOR (Fig. 5), so that RAPTOR directly participates in mTOR dimer stabilization.

The N-terminal helical solenoid of Tor is essential for dimerization and includes a distinctive elliptical spiral that can easily be seen in the 2D classes (Supplementary Fig. 2c). The middle of this elliptical spiral (which appears to be the most flexible portion of Tor, Supplementary Fig. 1b) is homologous with the region of mTOR that binds to the Tel2-containing chaperone complex, thus stabilizing the enzyme after it has been translated (residues 400–806 in human mTOR, 360–770 in KmTor)^44^.

Our structure also shows that the NRD inserted in the C-lobe of the kinase domain acts to restrict entry into the active site (Fig. 2b). Studies have shown that the deletion of the NRD activates mTOR^51^. This region was originally identified as the linear binding epitope for an antibody that activates mTOR^52^,^57^. The NRD, also known as the PIKK regulatory domain, is homologous with a region of ATR that binds to TopBP1, and TopBP1 was proposed to change ATR conformation, thereby increasing ATR's ability to bind substrates^58^. In DNA-PKcs, the PIKK regulatory domain was proposed to be a point of contact with Ku70/80 (ref. 58). Furthermore, mTOR residues Thr2446 and Ser2448 in the NRD are phosphorylated by p70-S6K1 (refs 59, 60). However, the effect of this phosphorylation on mTOR activity has remained elusive, since mutation of these residues to Ala or Glu did not affect mTOR activity *in vitro*^59^. The KmTor residue 2364, equivalent to human mTOR residue 2448, is at the outermost tip of the NRD.

This structure of a full-length Tor at sub-nanometer resolution indicates that the N-terminal half of the Tor polypeptide is formed from a complex topology of helical repeats, with two domains (spiral and cap) cradling the FATKIN (Fig. 1 and Supplementary Fig. 14). Although all of the PIKKs are likely to be composed of HEAT repeats, the low sequence similarity and variability in length of the N-terminal helical solenoids suggests that the architecture of these solenoids may not be conserved among the PIKKs. The variability of the solenoids is consistent with them being used by the PIKKs to interact with PIKK-specific adaptors and regulators. This insight into the Tor organisation will help to unravel its unique regulatory interactions. However, structures of Tor and other PIKKs at higher resolution are needed to fully understand the architectures and regulation of this family of large enzymes.

## Methods

### Engineering a *Kluyveromyces marxianus* strain overexpressing KmTor–Lst8 and RFP-inserted complexes

Unlike the genomes of *Saccharomyces cerevisiae and Schizosaccharomyces pombe*, the genome of *K. marxianus* codes for a single *TOR* homolog that also exists as such in its closely related yeast species *Kluyveromyces lactis*^61^. *K. marxianus* genomic DNA was purified from the *K. marxianus* wild-type strain (ATCC22296, LGC Standards Ltd., UK). The *K. marxianus* Tor and Lst8 coding fragments (KmTOR and KmLST8, respectively) were amplified by PCR from the purified *K. marxianus* genomic DNA. The KmTor fragment (4–4609 bp) was N-terminally fused with double Protein A (ZZ) tag with a linker sequence containing three TEV protease sites (multiple TEV sites sometimes gives better yield in cleaving from the IgG resin). The ZZ-linker-KmTor fragment was placed downstream of the strong galactose-inducible promoter (Somatogen Inc) with the natMX cassette fragment in pBluescript II KS(−) (pYO503). The full-length KmLST8 fragment was cloned between the galactose promoter and the MF(ALPHA)1 terminator. The GAL promoter-KmLST8-terminator cassette was then cloned into pBluescript II KS(−) already containing a kanMX cassette and the GAL4DB/VP16 transcriptional activator (pYO524). The plasmids were integrated into the *K. marxianus* genome by linearizing the plasmids using unique restriction enzyme sites (AflII and BstEII for pYO503 and pYO524, respectively).

For creating the strain overexpressing KmTor with an internal tandem RFP construct, a tandem tomato RFP coding fragment was first amplified by PCR and then inserted into the KmTor coding region between G323 and G324 of pYO503 by In-Fusion reaction (Clontech). The resulting plasmid (pYO853) was then linearized by AflII enzyme, and integrated into the Km genome. The resulting strain was used for integrating KmLST8 into the Km genome by linearizing the pYO524 plasmid with BsaAI.

For creating the plasmid with an internal tandem RFP insertion between M1190 and D1191 of KmTOR, the KmTOR fragment of pYO503 was replaced with a full-length KmTOR fragment including the 481 bp terminator region, then the tandem RFP fragment was inserted using PCR and In-Fusion reaction as described above (pYO1029). pYO1029 was linearized with the SacI enzyme and integrated into the Km genome of a strain, in which pYO524 had been already integrated. The plasmid with an internal tandem RFP insertion between T1175 and K1176 (pYO1036) was similarly made as pYO1029, and integrated into the Km genome in the same way as pYO1029.

### Protein expression

For the precultures, a couple of *K. marxianus* colonies were resuspended in 150 ml YEP (1.1% yeast extract, 2.2% bactopeptone and 55 mg l^−1^ adenine sulfate) and 2% (v/v) glucose and grown overnight shaking at 30 °C until fully saturated (OD_600_∼15). For protein induction, on the following day, 15 l of the main culture was prepared by diluting the pre-culture 1:100 (v/v) in the induction medium (YEP medium supplemented with 2% (v/v) galactose, 2% (v/v) glycerol and 3% (v/v) sodium lactate) and incubated for 24–27 h with shaking in 2-l-flasks at 220 r.p.m. and 30 °C.

### Purification of the KmTor–Lst8 complex

*K. marxianus* cells overexpressing the complex were harvested at 4 °C in an H-12,000 Sorvall rotor at 4,200 *g* for 15 min, then washed in ice-cold Millipore water followed by centrifugation in an H-6000 Sorvall rotor at 5,900 *g* for another 15 min. Subsequently, the wet pellets were resuspended in 2 volumes of the lysis buffer (50 mM Hepes pH 7.4 (23 °C), 150 mM KCl, 10% (v/v) glycerol, 0.3% (v/v) CHAPS, 0.5 mM TCEP, five tablets of cocktail inhibitors (EDTA-free, Roche)/0.5 l. 700 ml of the cell/buffer mixture was passed once through the ice-cold chamber of the cell disruptor (Constant Systems Ltd.,) applying a pressure of 35,000 p.s.i.

The lysate was cleared by centrifugation in a Ti45 rotor at 19,500 *g* for 13 min, the supernatant mixed with 3 ml IgG-resin slurry (IgG Sepharose 6 Fast Flow, GE Healthcare) and incubated at 4 °C for 1.5 h in 250 ml-conical bottles (Corning) on a roller at 16 r.p.m. After spinning the lysate in a Rotanta 460 swing-out rotor at 1,900 *g* for 2 min, the IgG-resin was collected and the supernatant carefully removed by pipetting. The resin was resuspended and transferred to a glass Econo-Column 2.5 × 10 cm (Bio Rad). All following steps were done at 4 °C, and a roller was used during the incubation steps. The lysis buffer was used throughout the IgG-affinity chromatography. The resin was first washed with two column volumes (CV) lysis buffer, followed by three washes with 2.5 mM ATP/25 mM MgCl_2_ in lysis buffer, with 5 min incubation between each wash. Again, the resin was washed with 2 CV lysis buffer, then treated with 30 μg RNase (R4875 Sigma) in 5 ml aliquots – repeated three times with 5 min incubation each. After a final wash with 6 CV buffer, the resin was resuspended in 12 ml lysis buffer and transferred to a 50 ml Falcon tube before adding 100 μg TEV protease and left rolling overnight. After cleaving, the protein was collected and diluted 1:1 with dilution buffer (50 mM Hepes pH 7.4 (23 °C), 10% (v/v) glycerol, 0.3% (v/v) CHAPS, 1 mM TCEP) before loading onto an anion-exchange column (Mono Q 0.8 ml, Pharmacia Biotech). Bound protein was eluted with a linear gradient from buffer A to buffer B (buffer A: 50 mM Hepes pH 7.4 (23 °C), 100 mM KCl, 0.3% (v/v) CHAPS, 1 mM TCEP; buffer B: 50 mM Hepes pH 7.4 (23 °C), 1 M NaCl, 0.3% (v/v) CHAPS, 1 mM TCEP). Finally, the protein was concentrated by ultrafiltration using a Vivaspin6 50,000 MWCO membrane concentrator to 2.5 mg ml^−1^. The same protocol was used for purifying the KmTor-RFP–Lst8 complexes.

### Cloning and purification of mouse mTOR(1360-2549) FATKIN

The mouse mTOR (1360–2549) FATKIN fragment was amplified by PCR from the *M. musculus* cDNA (IMAGE clone: 6465126). The mTOR fragment was fused with double protein A (ZZ) tag with a linker sequence containing three TEV protease sites. The ZZ-linker-mTOR fragment was placed downstream of a strong galactose-inducible promoter in the centromeric (CEN/ARS) plasmid pRS414 (pAB99). The pAB99 was transformed into *S. cerevisiae* BCy123 strain (MATa CAN1 ade2 trp1 Ura3-52 his3 leu2-3, 112 pep4::HIS3 prb1::LEU2 bar1::HisG lys2::pGAL1/10-GAL4). The BCy123 cells expressing mTOR FATKIN were grown in -TRP medium (8 g l^−1^ yeast nitrogen base, 11 g l^−1^ casamino acids, 55 mg l^−1^ adenine sulphate, 55 mg l^−1^ tyrosine, 55 mg l^−1^ uracil, 10 mg ml^−1^ leucine) supplemented with 2% glucose (v/v) at 30 °C overnight until saturated (OD_600_ ∼10). For protein production, the overnight culture was diluted 1:5 in the induction medium (-TRP medium supplemented with 2 % (v/v) galactose, 2% (v/v) glycerol and 3% (v/v) sodium lactate). Up to 15 l of culture were grown for 24–27 h with shaking in 2 l-flasks at 220 r.p.m. and 30 °C. The cells were harvested, disrupted and the mTOR FATKIN was affinity purified using the IgG resin as described for the KmTor–Lst8 complex. The mTOR FATKIN co-purified with the endogenous *S. cerevisiae* Lst8 (ScLst8). The elution pool containing the mTOR FATKIN-ScLst8 complex was finally purified using the gel filtration chromatography (Superdex200 10/30). The purified complex was concentrated to 1 mg ml^−1^ in 50 mM Tris pH 8.0, 300 mM NaCl, 0.3% (v/v) CHAPS, 1 mM TCEP using a Vivaspin6 50,000 MWCO concentrator. For the purpose of the kinase assay, 10% (v/v) glycerol was added to the mTOR FATKIN-ScLst8 complex before flash-freezing in the liquid nitrogen for storage at -80 °C.

### *In vitro* kinase assay

The kinase assay was done by following a published protocol for mTORC1 (ref. 32). The catalytic activity of KmTor–Lst8 (purified the same day and never frozen) or mouse mTOR FATKIN (1360–2549)-ScLst8 (expressed in *S. cerevisiae*, frozen, stored at −80 °C then thawed for the assay) was determined using 4 μg peptide substrate Sc4E-BP1 (GYDYSTTPGGTGRRRRR) (Cambridge Peptides Ltd., UK). The kinase reactions were carried out in a reaction buffer (25 mM Hepes pH 7.4 (23 °C), 50 mM KCl, 5 mM MnCl_2_, 5 mM MgCl_2_, protease cocktail inhibitor without EDTA (Roche)) in a final volume of 10 μl. The reactions were started with the addition of 100 μM ATP and 0.6 μCi (γ-^32^P)ATP, incubated for 15 min at 23 °C and stopped by addition of EDTA at a final concentration of 20 mM. Aliquots of 2.5 μl of each reaction were transferred onto a Grade P81 cation-exchange chromatography paper (GE Healthcare, Whatman). The membrane was dried in air for 5 min and then washed for 30 s in 30 ml 0.75% phosphoric acid. The procedure was repeated five more times with 5 min incubation each. Finally, the membrane was dried for 20 min at 40 °C. Subsequently, the membrane was exposed to a storage phosphor screen (GE Healthcare) and the ^32^P incorporation analysed using a Typhoon FLA 7000 phosphoimager. The same kinase reaction was done for a serially diluted enzyme with a fixed substrate concentration.

### Multi-angle light scattering

The mass in solution of the KmTor–Lst8 complex from *K. marxianus* was determined by size-exclusion chromatography coupled to multi-angle light scattering (SEC-MALS) measurements using a Wyatt Heleos II 18 angle light scattering instrument coupled to a Wyatt Optilab rEX online refractive index detector. The protein sample (100 μl of 1 mg ml^−1^) was resolved on a Superdex S200 10/300 analytical gel filtration column (GE Healthcare, UK) running at 0.5 ml min^−1^ in 50 mM Tris pH 8.8 (23 °C), 300 mM NaCl, 0.3% (w/v) CHAPS, 1 mM TCEP before passing through the light scattering and refractive index detectors in a standard SEC-MALS format. Buffers were filtered through a 0.22 μm pore size Stericup sterile filter (Millipore) before usage. The protein concentration was determined from the excess differential refractive index based on 0.186 RI increment for 1 g ml^−1^ protein solution. The concentration and the observed scattered intensity at each point in the chromatograms were used to calculate the absolute molecular mass from the intercept of the Debye plot using Zimm's model as implemented in Wyatt's ASTRA 6 software (commercially available from Wyatt technology, Santa Barbara, California).

### Cryo-EM grid preparation and microscopy

Aliquots of 3 μl freshly purified KmTor–Lst8 complex at a concentration of 2.5 mg ml^−1^ were added to a glow-discharged holey-carbon grids (Quantifoil Au R 0.6/1.0 or Au R 1.2/1.3, 300 mesh), blotted for 11–13 s at 4 °C and then plunge-frozen in liquid ethane. The cartridge-loaded grids were transferred into an FEI Titan Krios electron microscope operated at 300 keV. The micrographs were recorded on a FEI-Falcon II direct electron detector using an automated data acquisition software FEI-EPU at calibrated magnification of × 105,263, yielding a pixel size of ∼1.33 Å. Exposures of 40 e Å^−2^ over 1.5 s were dose-fractionated in 34 movie-frames. Defocus-range of 2.5–3.5 μm was used in the final data collection. The KmTor-RFP–Lst8 complexes were prepared for the cryo-EM analysis following the same protocol as already described for the KmTor–Lst8 complex. Micrographs for the KmTor-323RFP–Lst8 complex were recorded using a Tecnai F30 Polara transmission electron microscope operated at 300 keV with an FEI-Falcon III (ultra back-thinned) detector, under a calibrated magnification of × 78,000, pixel size of ∼1.34 Å and 3–4 μm defocus range. Micrographs for the KmTor-1175RFP–Lst8 and KmTor-1190RFP–Lst8 complexes were recorded using the Falcon II direct electron detector integral to a Titan Krios transmission electron microscope operated at 300 keV under calibrated magnification of × 105,263, pixel size of ∼1.34 Å and 3–4 μm defocus range. We used total of 40 or 35 e Å^-2^ over 1.5 s exposures that were dose-fractionated in 34 or 25 movie frames, respectively. The data collection and processing are summarized in Supplementary Table 1.

### Image processing

An initial data set was collected at a nominal magnification of × 47,000 (calibrated magnification— × 81,395) resulting in ∼1.72 Å/pixel sampling with a 4 s exposure. From 44 micrographs, 3,175 particles were subjected to 2D classification in EMAN2 (ref. 62) and distinct views from these classes were used to generate an initial model with a C2 symmetry imposed. The model that matched the class averages best was then used for refinement in RELION. The initial refinement of ∼38,000 particles including frames 1–32 (last 36 frames were discarded) from this low magnification data yielded a map of about 8.5 Å. The human mTOR FATKIN–mLST8 model (PDB ID 4JSV)^37^ was fitted in our cryo-EM map to reveal the correct hand that was then used in further refinements.

We then collected three additional data sets with a total of 2,332 cryo-EM micrographs (Supplementary Table 1). The movie frames for each micrograph were corrected and summed for the whole-image drift using MOTIONCORR^63^. The GPU-accelerated program Gctf (http://biorxiv.org/content/biorxiv/early/2015/07/14/022376.full.pdf, pre-released version created by Dr Kai Zhang, MRC-LMB) was used to determine contrast transfer function parameters from the resulting drift-corrected average micrographs. All subsequent data processing steps were performed in RELION^64^ (version 1.4-beta), if not mentioned otherwise.

Initially, 1,500 manually selected particles were used for reference-free 2D-classification. The resulting 2D-class averages were low-pass filtered to 20 Å and then used as templates for the reference-based auto-picking of the entire data set. The automated particle-selection was examined manually for each micrograph to remove ice spots and contaminants before extracting the sum of 197,535 particles. To clear the particle selection from false positives^65^, we used reference-free 2D class averaging that yielded 89,430 particles from good 2D-classes that were then subjected for 3D-processing.

3D-classification with 10 classes was done by imposing the C2 symmetry, using finer angular sampling of 3.7°, regularization parameter T of 2 and without performing local angular search around refined orientations during 25 iterations. This resulted with one 3D-class average containing 28,877 particles that stood out with the relatively well-reconstructed features of the kinase domain and the helical solenoid. Auto-refinement of this particular class gave reconstruction at an overall resolution of 6.7 Å.

We used statistical movie processing in RELION^64^,^66^ to estimate per-particle beam-induced movements for the first 32 movie-frames with running averages of seven movie-frames and a standard deviation of 1 pixel for the translational alignments. We then used particle-polishing^67^ step that fits linear tracks through the estimated particle movements to complete the beam-induced movement modelling. This step also included the temperature/B–factor weighting for estimating the radiation-damage that is dose and resolution-dependent using the reconstructions by running averages of three movie-frames.

The polished particles (movement-corrected) were used for the auto-refinement with a soft auto-mask (12 pixel fall-off) around the entire map that resulted in a final map with an overall resolution of 6.1 Å. Reported resolutions are based on the gold-standard FSC=0.143 criterion^68^ and the Fourier Shell Correlation (FSC) curve was corrected for the effects of a soft mask on the FSC curve using high-resolution noise substitution^69^. This cryo-EM map was used for interpretation and model building. Before visualization, all density maps were corrected for the modulation transfer function of the detector and then sharpened by applying a negative B-factor that was estimated using automated procedures^70^. The ResMap^71^ was used for estimating local resolution variations.

### Building a model into the density

A model without side chains was built into the EM density using Coot^72^. For the FATKIN, the human mTOR structure (PDB ID 4JSV) was used without side chains as a starting model^37^. There was no initial model for the N-terminal helical solenoid. A previous HHPRED sequence analysis had suggested that the region is made up of HEAT repeats^53^. Inspection of the density suggested a collection of paired helices characteristic of HEAT repeats. We established two landmarks in the density of the N-terminal helical solenoid by constructing two variants of the protein, with each variant having a tandem RFP module inserted into it. Two points of insertion were chosen, thereby splitting the N-terminal solenoid into three regions of roughly similar sizes. The number of helices predicted by the HHPRED study^53^ closely matched the actual number of tubular helical densities in the cryo-EM density for the N-terminal solenoid (there are 60 helices apparent in the density, consistent with 60 predicted for yeast Tor1 and 64 for human mTOR). Idealized helices were built into each of these tubes and assigned approximate residue numbers based on the HHPRED analysis and the two RFP landmarks. In some cases, the density connecting one helix to the next in the solenoid was also clearly visible, however, in many cases a few residues were missing in these loops. The model has no side chains. All newly modeled regions were built using only atoms present in ALA and GLY, however, approximate residue numbers have been assigned throughout, with appropriate residue types. Although the sequence register is approximate, we have assigned it throughout to help facilitate use of the structure. The register of the sequence to the density is likely best in the FATKIN, for which there was a good starting model^37^. There are 328 residues missing out of 2,450 in the final model of KmTor. At the N-terminus, there are 63 residues not resolved, with no residues missing at the C-terminus. Almost all of the missing residues are in loops between helices. The longest internal stretch of missing residues is 17 (residues 867–883 in the linker leading to the bridge and residues 1670–1686 in TRD3 of the FATKIN), and the average stretch of internal missing residues is six residues. The density for Lst8 was difficult to interpret, since it is entirely *β*-strands connected by loops. The model for Lst8 was an homology model based on the mLST8, and this was not substantially altered in manual building. The model building for each domain is summarized in Supplementary Table 2.

The model was refined using PHENIX real-space-refine^73^. The final model has 80% of residues in the most-favored regions of the Ramachandran plot with 0.65% outliers. The model was converted into density by EMAN2 (ref. 62). The FSC curve of the model versus map was calculated using the Xmipp package^74^ with 7.1 Å resolution based on the FSC=0.50 criterion (Supplementary Fig. 1a).

### Data processing for RFP-inserted complexes

We have processed the KmTor-323RFP–Lst8, KmTor-1175RFP–Lst8 and KmTor-1190RFP–Lst8 data sets in the same way as already described for the KmTor–Lst8, however, without the final motion correction. For the 3D classification we have used the KmTor–Lst8 reference model low-pass filtered to 60 Å. After refinement, we post-processed the maps by RELION to obtain the final 8.5 Å maps with weak densities for the RFP tags (Supplementary Fig. 16a).

Because the density of the flexible tandem RFP tag (54 kDa) inserted in the KmTor is weak, we performed masked classification on only one of the two tandem tags in the dimer with subtraction of the signal from the rest of the KmTor-RFP–Lst8 complex^75^. Even though the tandem RFP tag is present in each of the C2 symmetry-related KmTor molecules, we have assumed that the tags exhibit independent motions. Therefore, before the signal subtraction, we have first rotated each refined KmTor-RFP–Lst8 particle by 360/2° and then combined all the rotated particles with the original ones. This way, we ensured that all the RFP tags are put in the same position. We were then able to independently perform masked classification assuming no symmetry on the sets of about 36,000 (KmTor-323RFP–Lst8), 15,000 (KmTor-1175RFP–Lst8) and 25,000 (KmTor-1190RFP–Lst8) particles, with the subtracted signal, yielding three major classes that showed good density for the RFP tag. During classification, we did not perform particle alignment but kept all the orientations fixed at the values determined in the original refinement. The original particles without subtracted signal from these three good classes were then independently subjected to separate 3D-auto refinement runs, without imposing C2 symmetry but using a mask around one RFP tag and the KmTor–Lst8 complex. After post-processing we obtained a 9 Å map from 10,400 KmTor-323RFP–Lst8 particles, a 10.3 Å map from 5,253 KmTor-1175RFP–Lst8 particles and a 10.5 Å map from the 5,200 KmTor-1190RFP–Lst8 particles (Supplementary Fig. 16c). Taking the approach of masked classification with the signal subtraction we were able to obtain a prominent density for 323 tandem RFP, 1175 tandem RFP and 1190 tandem RFP on one Tor molecule of the KmTor–Lst8 complex.

## Additional information

**Accession codes:** EMDB accession code EMD-3329 (KmTor-Lst8), EMD-3334 (KmTor–323RFP-Lst8), EMD-3335 (KmTor–1190RFP-Lst8), EMD-3336 (KmTor-1175RFP-Lst8) and PDB ID 5fvm (KmTor–Lst8).

**How to cite this article**: Baretić, D. *et al*. Tor forms a dimer through an N-terminal helical solenoid with a complex topology. *Nat. Commun.* 7:11016 doi: 10.1038/ncomms11016 (2016).

## Supplementary Material

Supplementary Information Supplementary Figures 1-16, Supplementary Tables 1-2 and Supplementary References
