# Supplementary material for: Tor forms a dimer through an N-terminal helical solenoid with a complex topology
Source: Nat Commun. 2016 Apr 13;7:11016. doi: 10.1038/ncomms11016 (PMC4833857; doi:10.1038/ncomms11016)
Supplement: Supplementary Information — Supplementary Figures 1-16, Supplementary Tables 1-2 and Supplementary References [file ncomms11016-s1.pdf]

Supplemental Information.

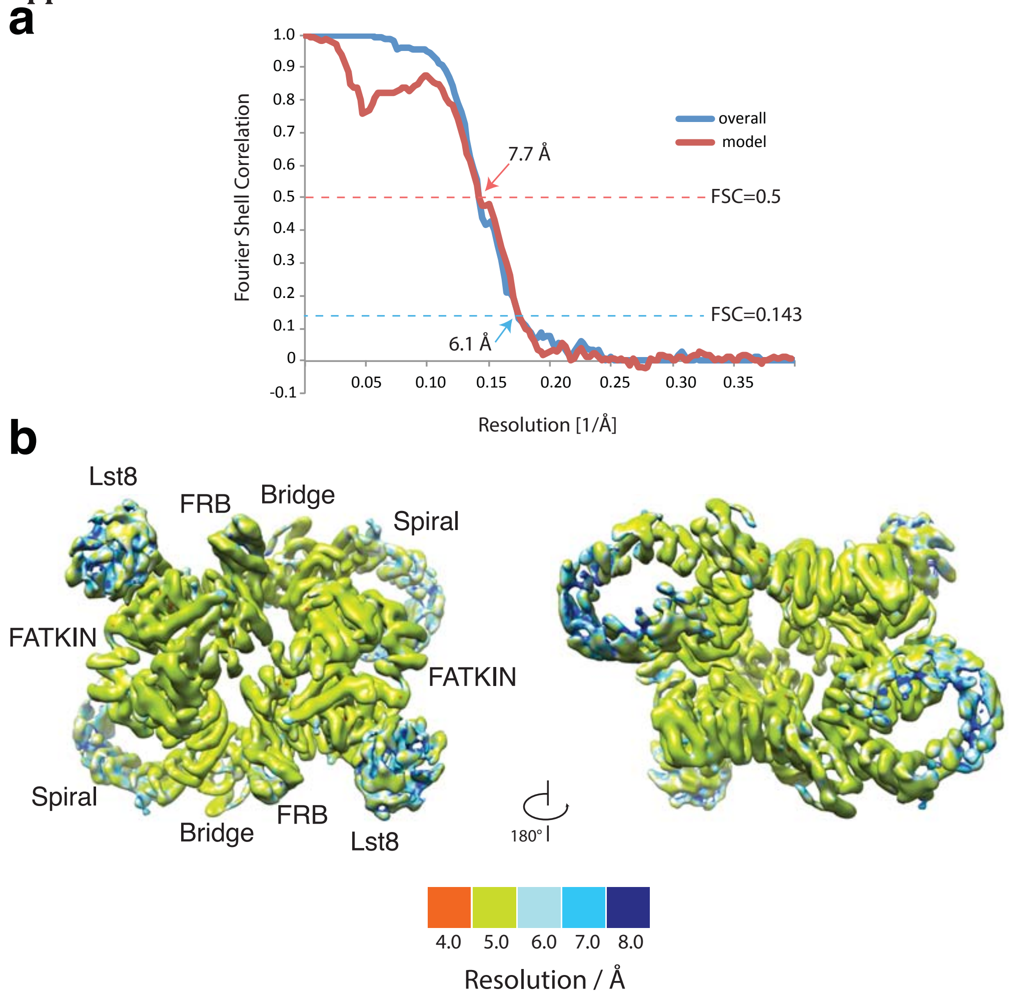

Supplementary Figure 1 | Resolution of the 3D reconstruction.

a. Gold standard Fourier shell correlation (FSC) (blue) and FSC comparing the model with the sum of the two half-maps used for the gold-standard FSC (red)[1].

b. Local resolution of the KmTor–Lst8 cryo-EM density map as calculated by the program ResMap[2]. Positions of the domains of the KmTor–Lst8 complex are indicated in the cryo-EM map on the left.

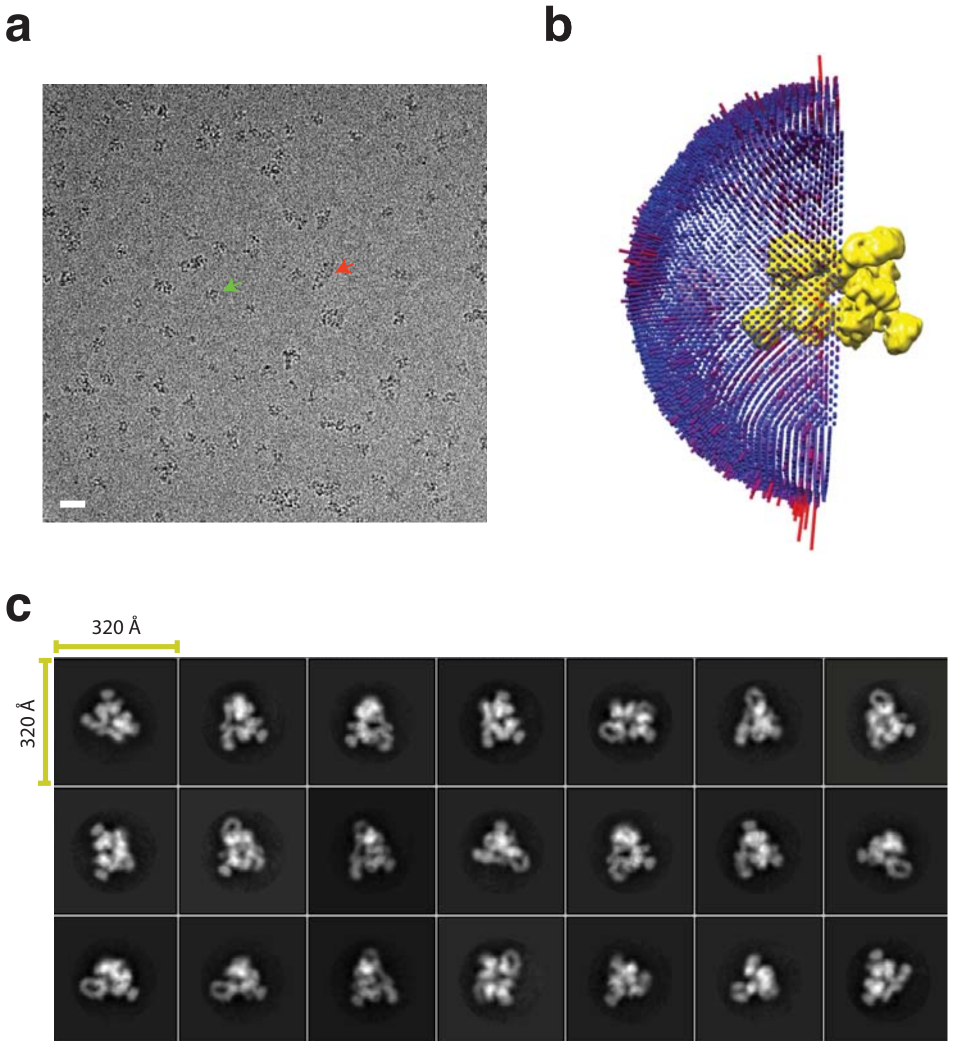

Supplementary Figure 2 | Cryo-EM reconstruction of the KmTor–Lst8 complex.

a. An example micrograph of KmTor–Lst8 particles in vitrified ice. A green arrow indicates a typical dimer. The red arrow points to an example of larger assemblies that were excluded from the reconstruction. Scale bar, 40 nm.

b. Euler angle distribution of all particles included in the final 3D-volume reconstruction with respect to the reference volume assigned by RELION[3]. The heights of the spikes emerging from a half-sphere relate to the fraction of particles with a given orientation visualised with UCSF Chimera[4] (Cylinders are

colored continuously from blue to red based on the Z score for a given orientation in the probability distribution. Brightest red orientations are the most common orientations).

c. Typical 2D class averages included in the 3D-volume reconstruction with a scale bar.

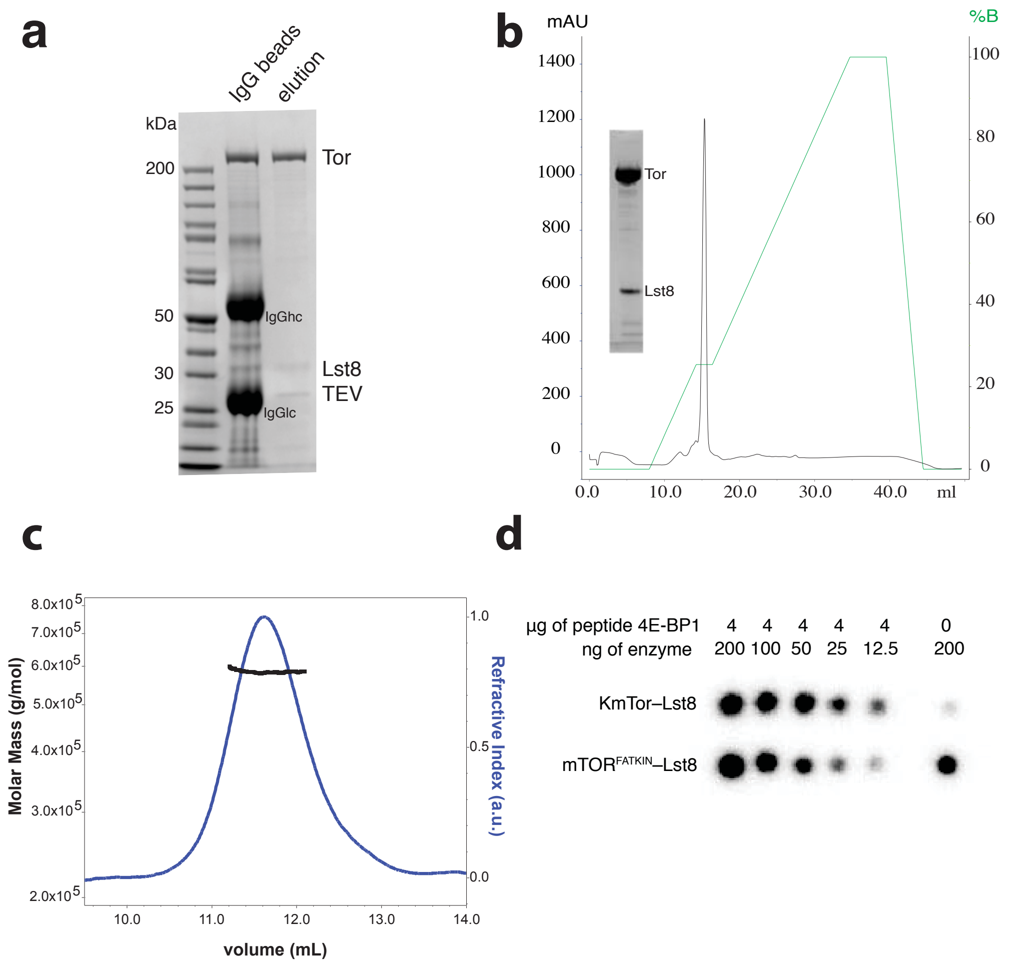

Supplementary Figure 3 | A summary of the purification of KmTor–Lst8.

a. Affinity purification of the double protein-A-tagged KmTor–Lst8 complex on IgG resin (SDS Coomassie-stained gel): MW standards (lane 1), Protein bound to the IgG beads, with IgG chains present (heavy, IgGhc and light, IgGlc) (lane 2), TEV-cleaved protein eluted (lane 3).

b. Anion chromatography of the KmTor–Lst8 complex. The inset shows an SDS Coomassie-stained gel of the peak fraction. The absorbance (mAu) at 280 nm is indicated by the black trace. The percent buffer B is indicated with the green trace.

c. Analysis of the mass of the purified KmTor–Lst8 complex by SEC (size-exclusion chromatography) using an S200 column coupled to MALS (Multi-angle light scattering) detectors. The analysis suggests that the material has an average mass of 583 kDa. The expected mass of a dimer of the KmTor–Lst8 heterodimer is 624 kDa. This indicated a larger discrepancy than we would expect based on the typical accuracy of SEC MALS, which is on the order of 2-3% of particle mass, as observed for the bovine serum albumin (BSA) standard. The sample was run at a relatively low concentration where we have plenty of light scattering signal, but a less accurate estimate of the concentration, which would limit the accuracy of the mass determination.

Close inspection of the SEC MALS chromatogram shows a slight shoulder at longer retention times. This has lower mass than that of the main peak analysed. Due to the overlap between this and the main peak, the mass determined will be the weighted sum of the masses of the species present. This shoulder is a minor component of the total protein eluting and may represent some low level of dissociated material in equilibrium with the main stable complex.

d. The KmTor–Lst8 complex is catalytically active. Phosphorylation of a 4E-BP1 substrate peptide as a function of enzyme concentration, as visualized by ^{32}P autoradiography. The activity of the mouse mTOR FATKIN is shown for comparison. The mouse mTOR shows significant autophosphorylation (0 ng peptide), while KmLst8 shows very little.

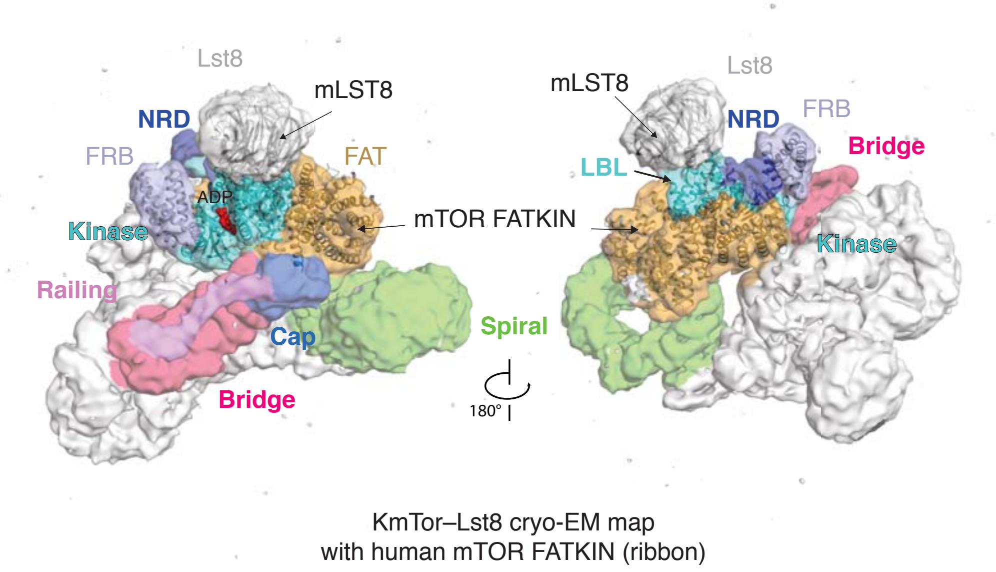

Supplementary Figure 4 | The human mTOR FATKIN/mLST8 placed into the EM density of KmTor–Lst8.

The KmTor–Lst8 density is shown contoured at 3.0σ so that the railing density is apparent. One monomer in the cryo-EM map of a dimer of heterodimers is colored by domain. The human mTOR FATKIN (PDB ID 4JSV, shown as a ribbon) fits well into the KmTor density with no adjustments. The FATKIN constitutes about one half of the KmTor structure.

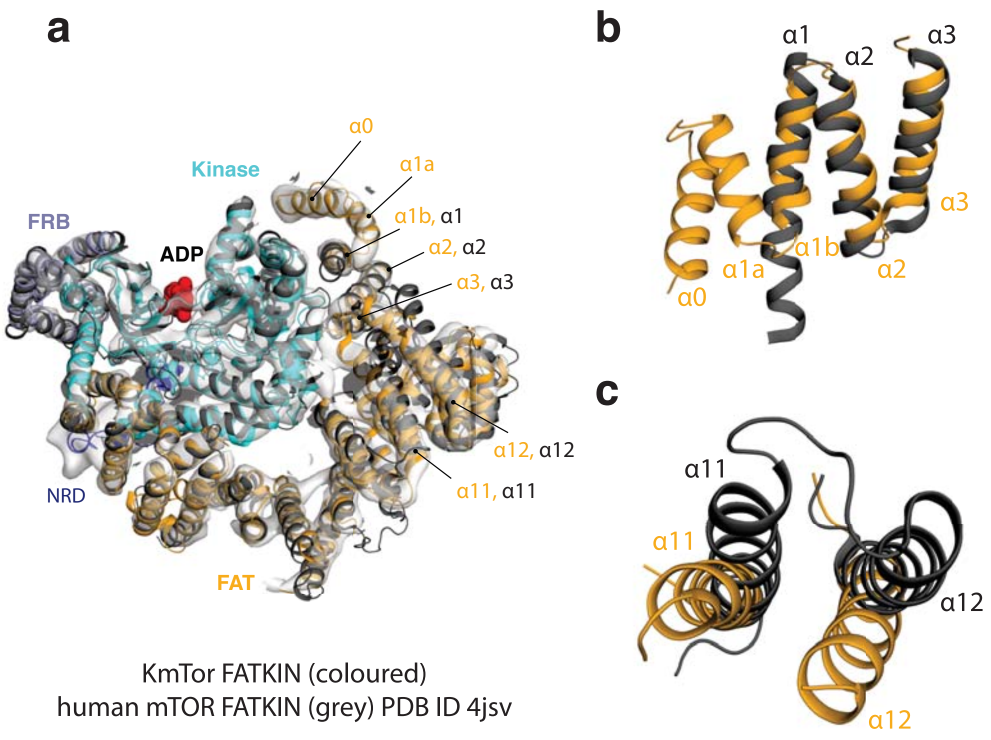

Supplementary Figure 5 | Comparison of the KmTor FATKIN with the human mTOR FATKIN.

a. Ribbons representing the KmTor FAT and kinase domains are colored orange and cyan, respectively. The human mTOR FATKIN (PDB ID 4JSV) is superimposed and shown as a black ribbon. The KmTor EM density is shown contoured at 11σ. The NRD density (blue) is contoured at 2.5σ.

b. The KmTor TRD1 is extended by two helices (α0 and α1a) relative to human mTOR (PDB ID 4JSV).

c. Helices α 11 and α 12 in TRD2 are shifted in KmTor relative to human mTOR (PDB ID 4JSV).

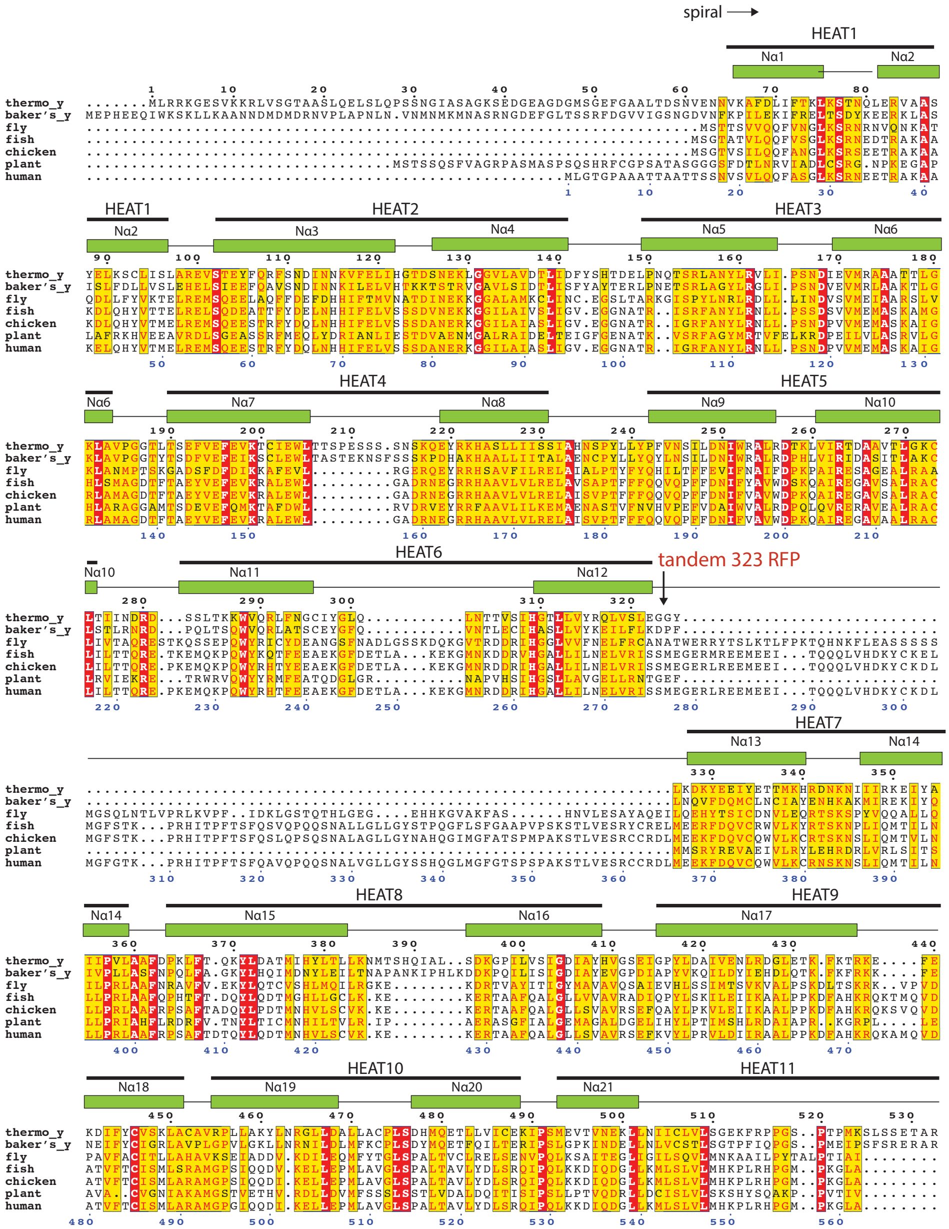

Supplementary figure 6

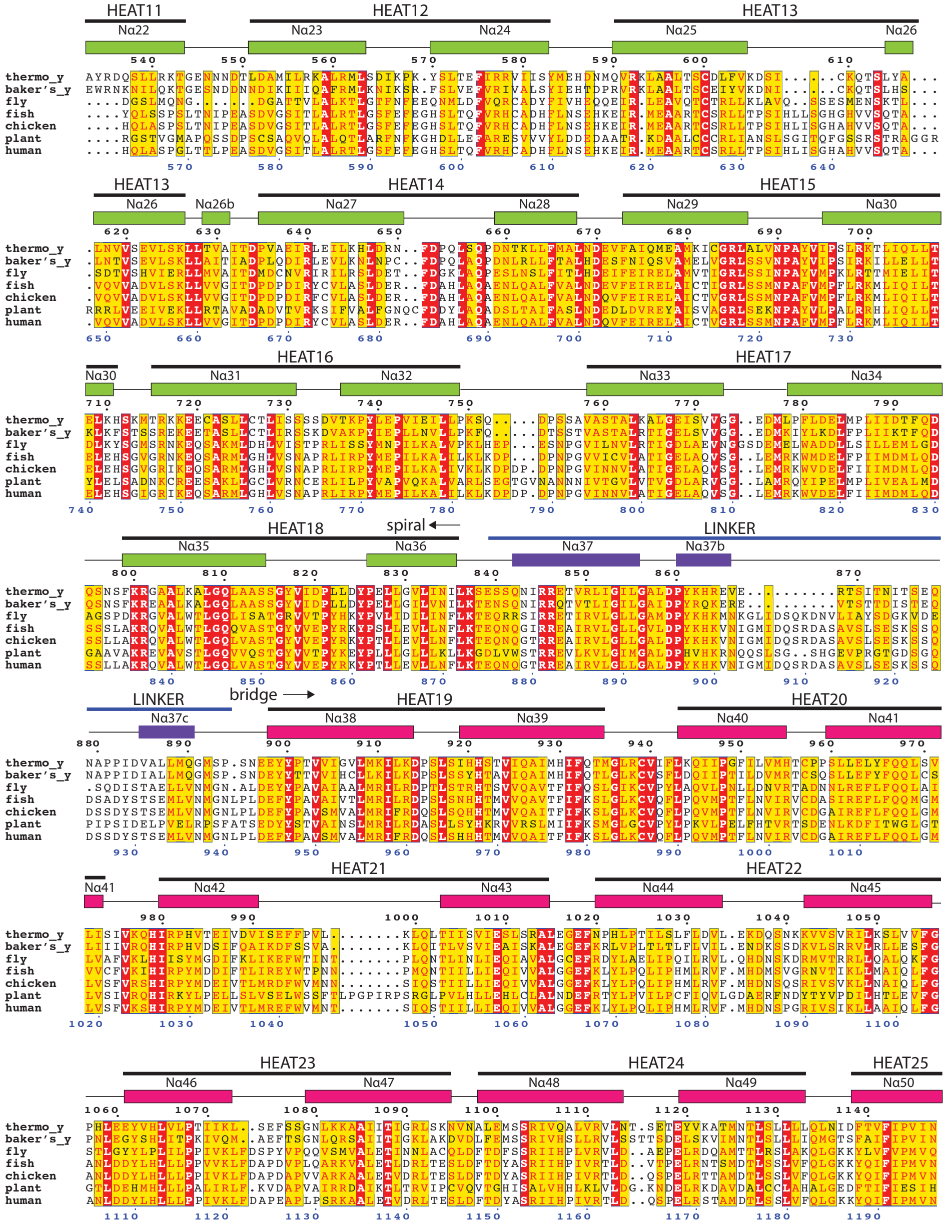
HEAT11
HEAT12
HEAT13
HEAT14
HEAT15
HEAT16
HEAT17
HEAT18
spiral
LINKER
bridge
HEAT19
HEAT20
HEAT21
HEAT22
HEAT23
HEAT24
HEAT25
thermo_y
baker's_y
fly
fish
chicken
plant
human

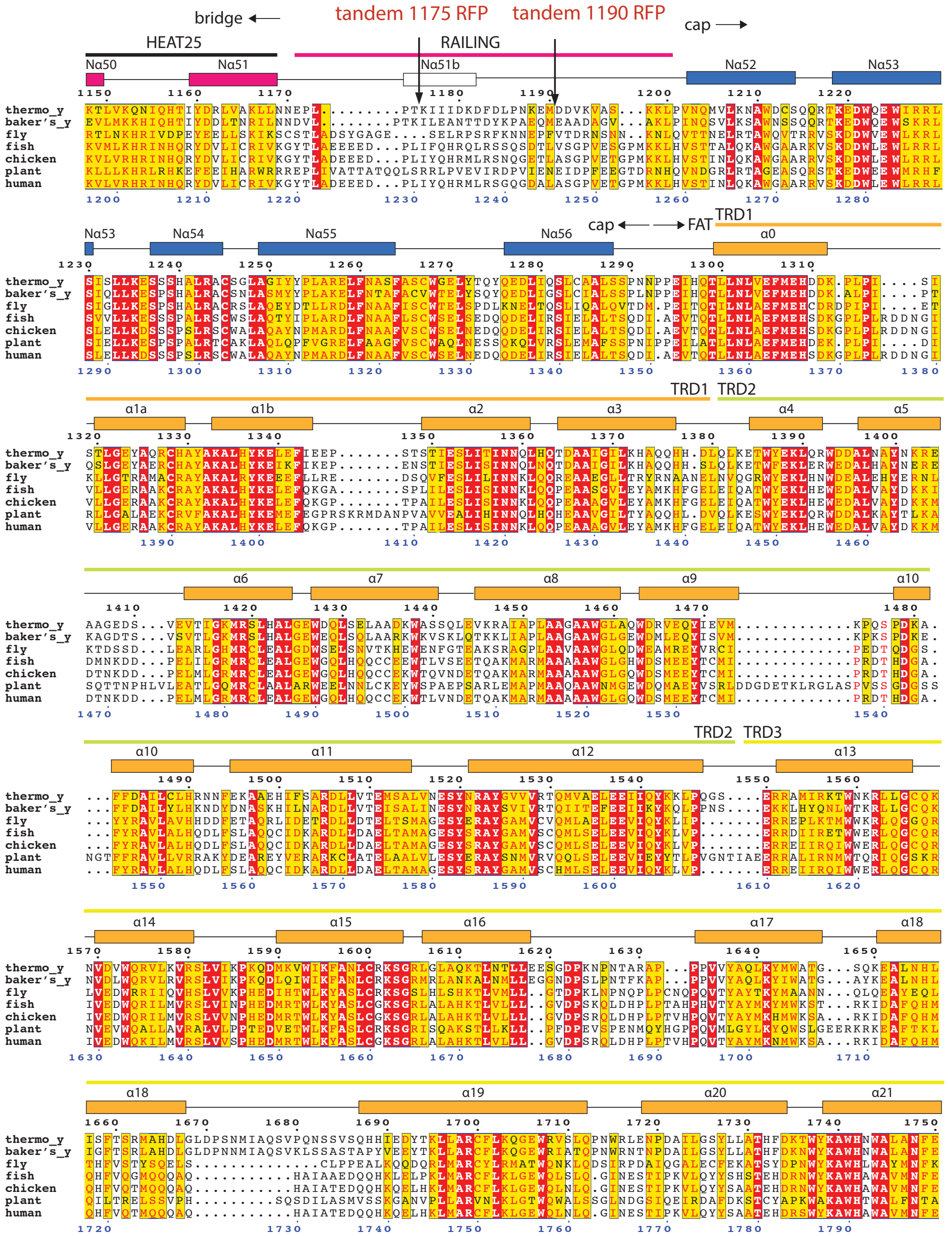
bridge
tandem 1175 RFP
tandem 1190 RFP
cap
HEAT25
RAILING
cap
FAT
TRD1
TRD1
TRD2
TRD2
TRD3
thermo_y
baker's_y
fly
fish
chicken
plant
human

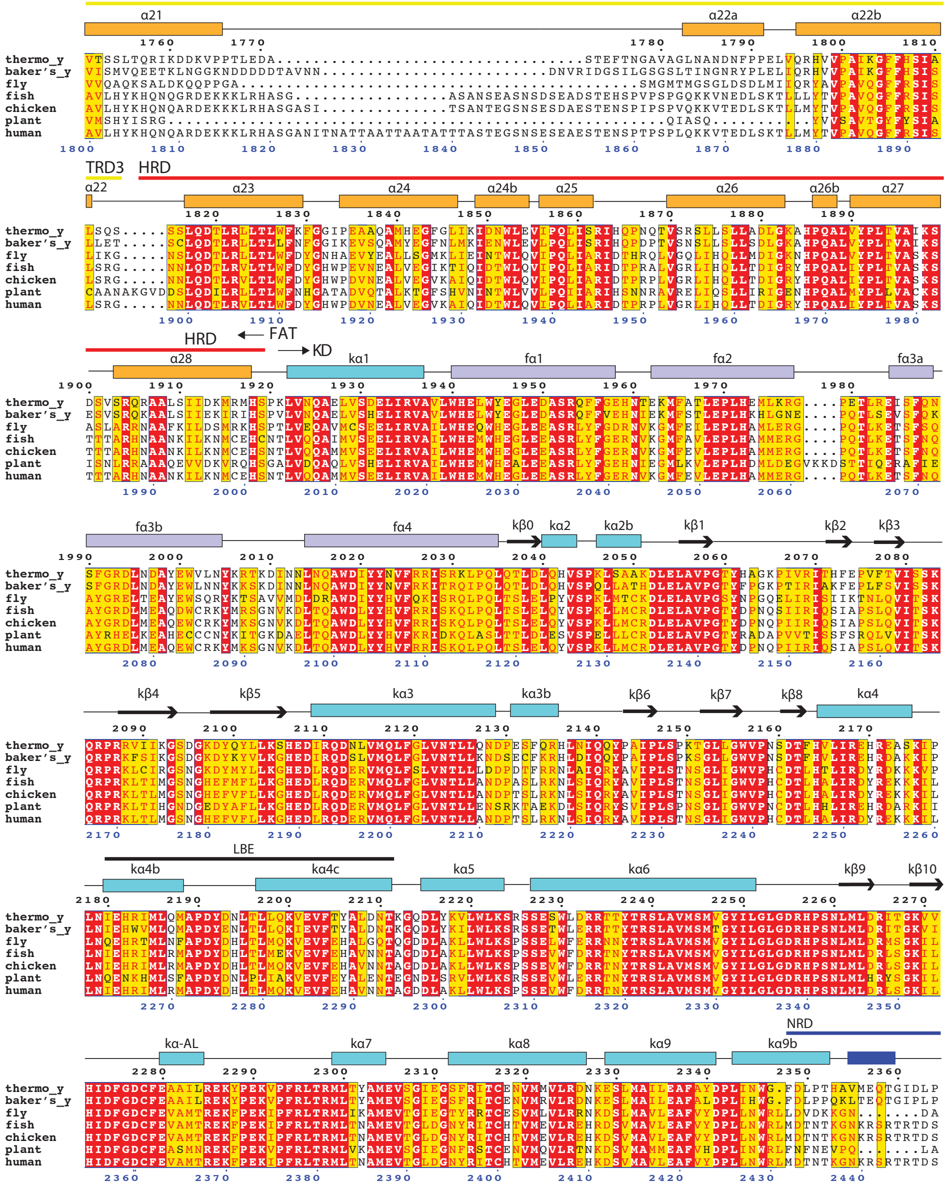
TRD3
HRD
FAT
KD
LBE
NRD
thermo_y
baker's_y
fly
fish
chicken
plant
human

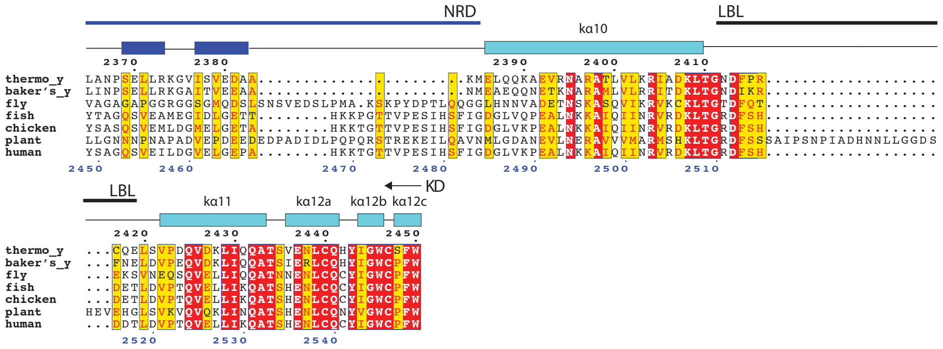

Supplementary Figure 6 | Multiple sequence alignment of TORs and mTOR.

The sequence of thermotolerant *K. marxianus* Tor determined and used in the course of this study is shown on the first line (thermo_y). The numbering at the top refers to KmTor. Blue numbering at the bottom refers to human mTOR (*H. sapiens*, 2549 a.a., UniProt P42345.1). Secondary structure elements are shown above the sequence in colours that correspond to the domains of KmTor as illustrated in Fig. 1. Twenty-five KmTor HEAT repeats are indicated above the secondary structures. In addition, sequences of baker's yeast (baker's_y, *S. cerevisiae*, 2470 a.a., SGD YJR066W), fruit fly (*D. melanogaster*, 2470 a.a., UniProt Q9VK45.1), fish (*D. rerio*, 2515 a.a., GenBank NP_001171773.1), chicken (*G. gallus*, 2521 a.a., NCBI XP_417614.3) and plant (*A. thaliana*, 2481 a.a., GenBank NM_103891.3) orthologs are shown. The sequences were fetched from repositories (above) and aligned in MUSCLE[5]. The alignment was then manually edited in Jalview 2[6]. The sequence alignment was rendered in ESPript 3.0[7]. The fully conserved residues are in white letters against red background, while scored as similar are represented by letters on yellow background. Secondary structure elements (α-helices shown as bars and β-strands as arrows) and defined domains/regions boundaries were created manually.

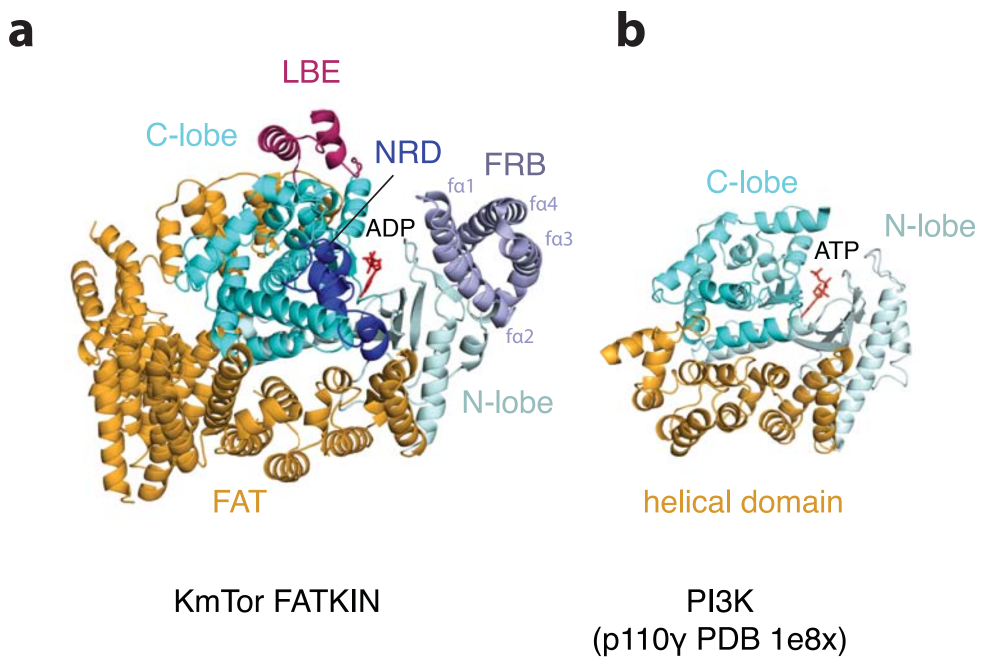

Supplementary Figure 7 | The KmTor FATKIN has a kinase domain that is similar to phosphoinositide 3-kinases (PI3K).

The KmTor kinase domain (cyan, left) is similar to the PI3K kinase domain (cyan, right). However, the KmTor kinase domain bears three elaborations that restrict access to the active site (shown with an ADP modelled into it, red); the Lst8-binding element (LBE), negative regulatory domain (NRD) and FKBP12-rapamycin-binding domain (FRB). The KmTor FAT domain (orange) includes a HEAT-repeat domain (HRD) that is roughly similar to the helical domain of PI3K (orange).

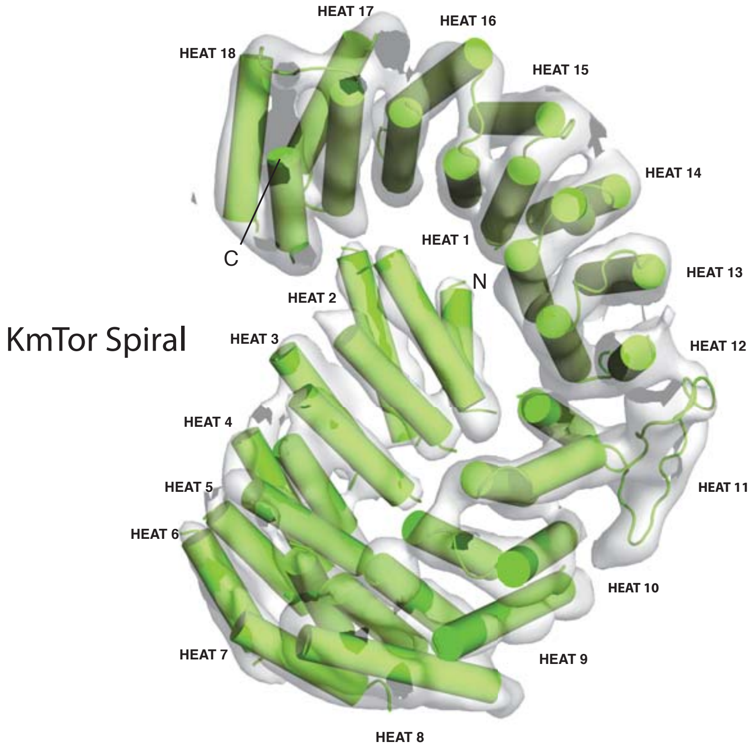

Supplementary Figure 8 | The spiral domain of the KmTor.

The structure of the spiral domain model (in cylinder representation) superimposed on the KmTor–Lst8 cryo-EM density contoured at 10σ. The spiral is made up of helical pairs that form 18 HEAT repeats. Numbering of the HEAT repeats is as indicated on the sequence alignment (Supplementary Fig. 6).

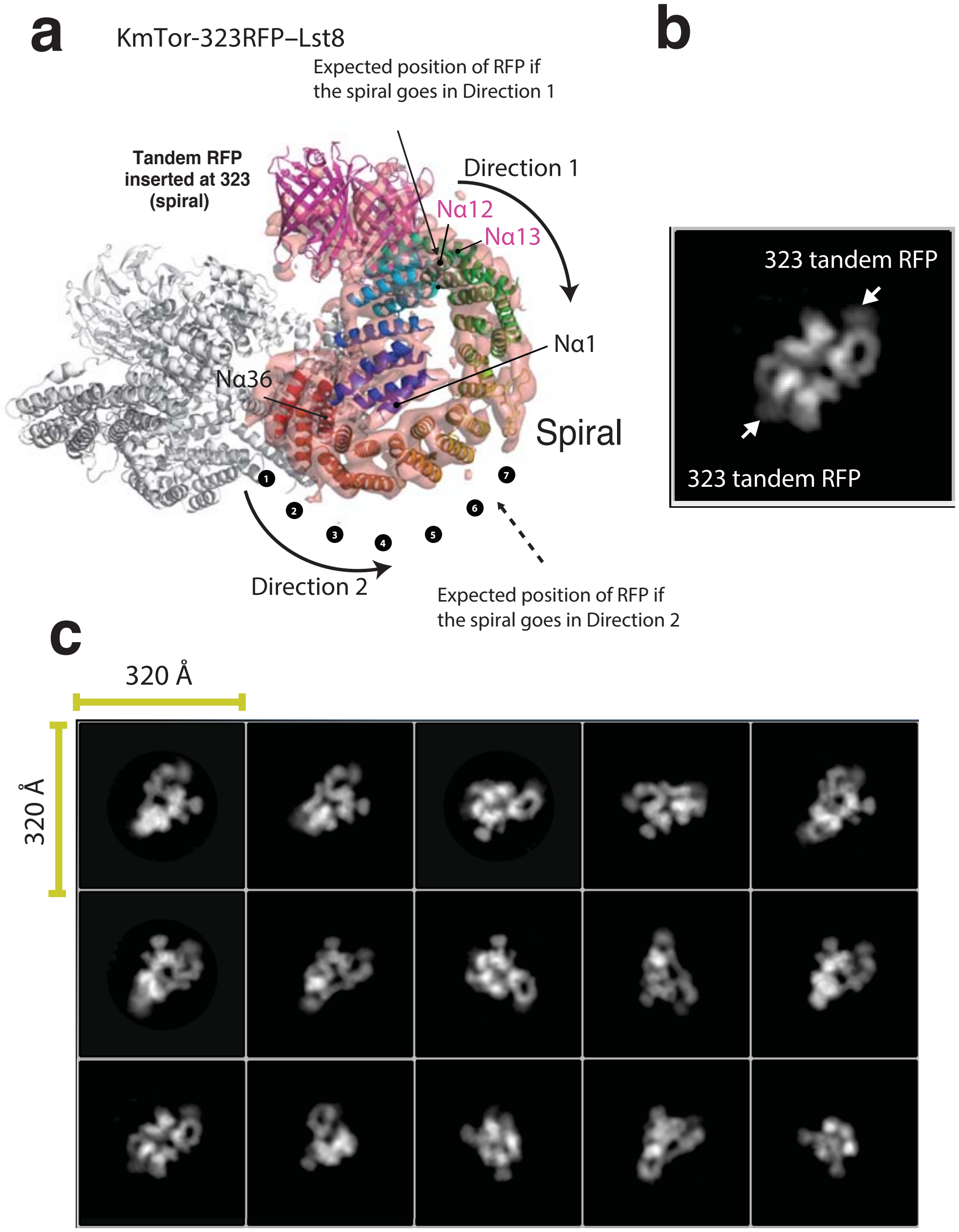
a
KmTor-323RFP–Lst8
Expected position of RFP if the spiral goes in Direction 1
Tandem RFP inserted at 323 (spiral)
Direction 1
Nα12
Nα13
Nα1
Nα36
Spiral
1
2
3
4
5
6
7
Direction 2
Expected position of RFP if the spiral goes in Direction 2
b
323 tandem RFP
323 tandem RFP
c
320 Å
320 Å

d

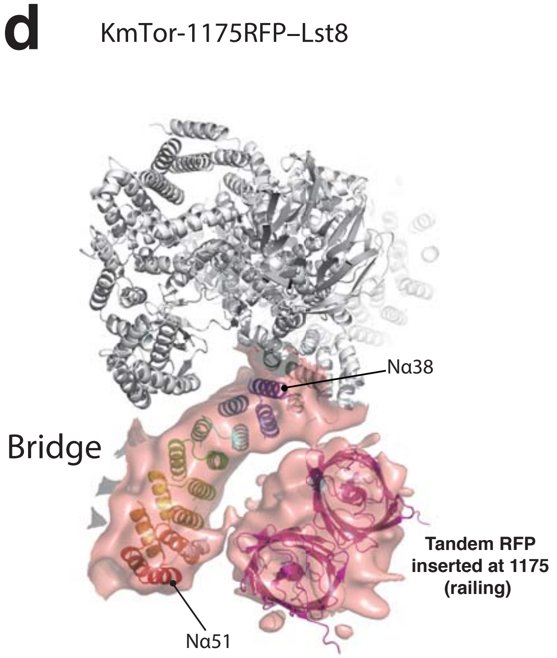
KmTor-1175RFP–Lst8
Nα38
Bridge
Tandem RFP
inserted at 1175
(railing)
Nα51

e

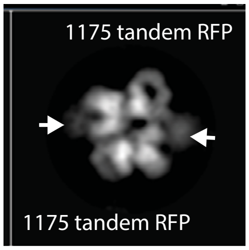
1175 tandem RFP
1175 tandem RFP

f

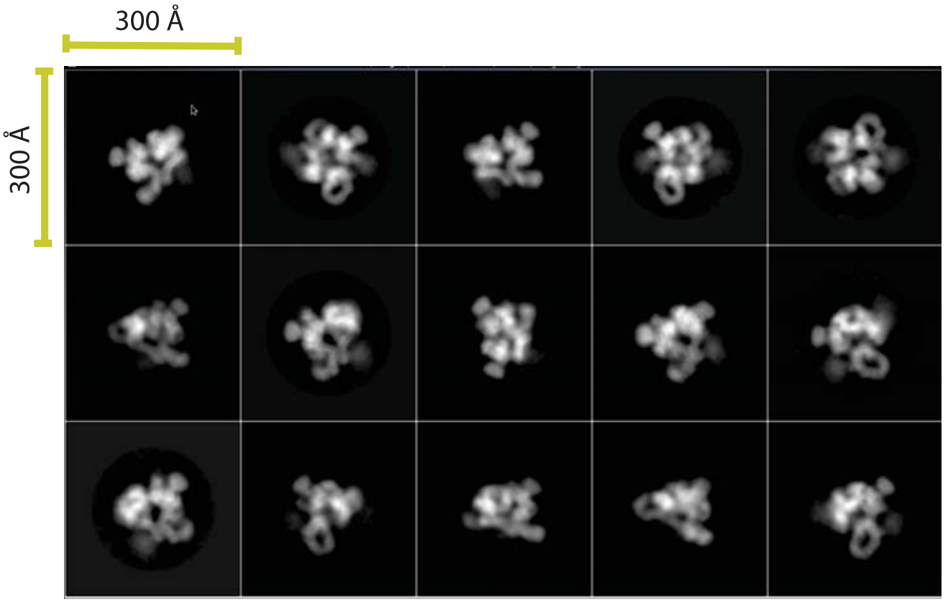
300 Å
300 Å

g

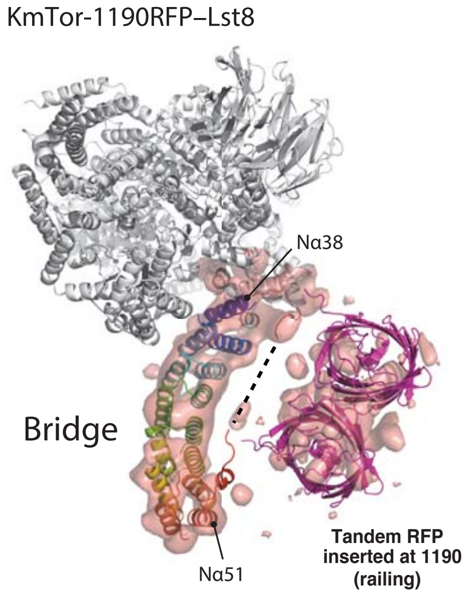

h

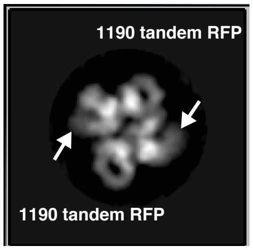

i

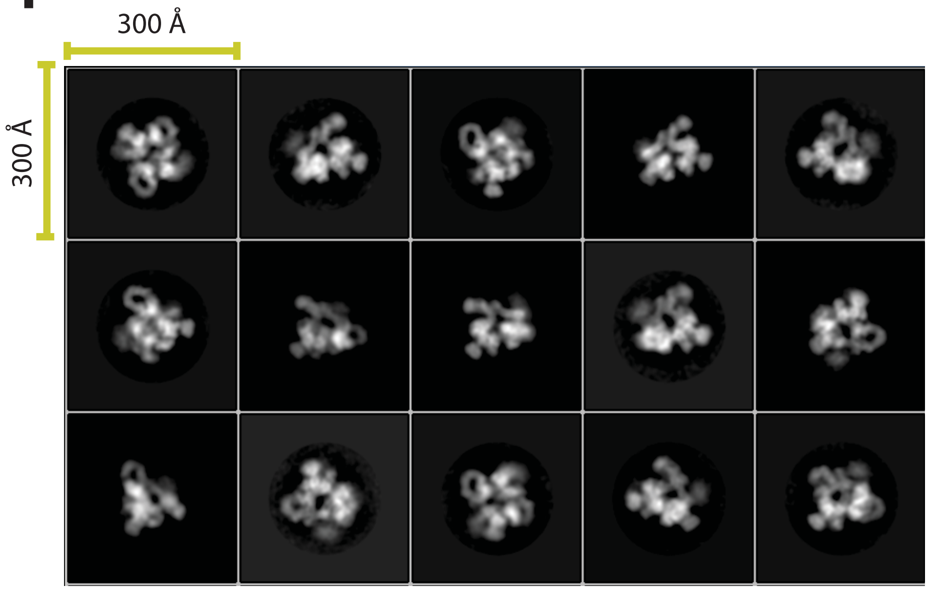

Supplementary Figure 9 | RFP insertion landmarks to determine the direction of the N-terminal solenoid.

a. For clarity, only a KmTor monomer is shown, and only the cryo-EM KmTor-RFP density associated with the spiral is illustrated. To determine the N- to C-direction of the spiral, tandem RFP was inserted between Tor residues 323 and

324 (between helices Nα12 and Nα13, KmTor-323RFP–Lst8). If the direction of the spiral is Direction 1 (N-terminus free and the C-terminus against TRD2), then the RFP would be at the indicated position. This is where we find the RFP density. If the direction of the spiral is Direction 2 (N-terminus buried and C-terminus free), the RFP would be at a very different position. The numbering (1-7) represents the HEAT repeats counted from the beginning of the polypeptide if the spiral goes in Direction 2. In this case, the tandem RFP would be between the repeats 6 and 7. Clearly, Direction 1 is correct, and the spiral is colored blue to red from N-terminus (Nα1) to C-terminus (Nα36). A model of tandem RFP (PDB ID 1GGX)[8] is fitted to the RFP density.

b. A 2D class average for the KmTor-323RFP–Lst8 complex. Protuberances on the spiral as a result of the tandem RFP insertion are indicated with arrows.

c. A selection of the 2D class averages of KmTor-323RFP–Lst8.

d. A tandem RFP inserted between KmTor residues 1175 and 1176 in the railing. The cryo-EM map is depicted for the bridge and the tandem RFP.

e. A 2D class average for KmTor-1175RFP–Lst8. Protuberances on the railing as a result of the tandem RFP insertion are indicated with arrows.

f. A selection of the 2D class averages of KmTor-1175RFP–Lst8.

g. A tandem RFP insertion between residues 1190 and 1191 in the railing, and the cryo-EM map of the KmTor-1190RFP–Lst8 is shown. A dashed line indicates the path of the railing in the higher resolution parent KmTor–Lst8 structure. The bridge is colored blue to red from its N- (Nα38) to its C-terminus (Nα51).

h. A 2D class average for KmTor-1190RFP–Lst8. Protuberances on the railing as a result of the tandem RFP insertion are indicated with arrows.

i. A selection of the 2D class averages of KmTor-1190RFP–Lst8.

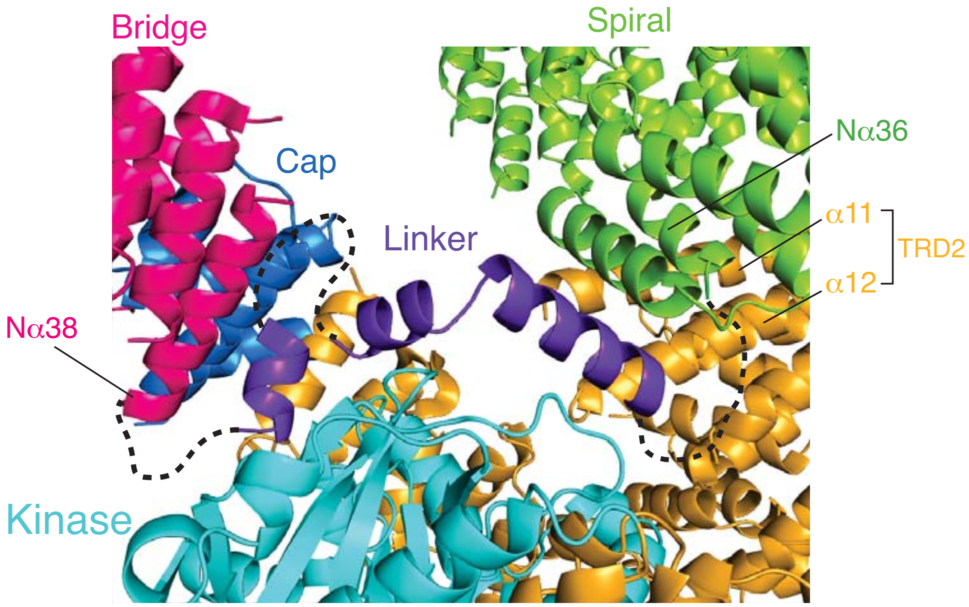

Supplementary Figure 10 | Linker and the Spiral/TRD2 interface.

A close view of the linker between the C-terminus of the spiral (Nα36, green) and the N-terminus of the bridge (Nα38, purple). Dashed curves indicate loops that are not present in the KmTor model. The C-terminus of the spiral packs against TRD2 (α11 and α12) of the KmTor FATKIN. Domain colors as in Fig. 1.

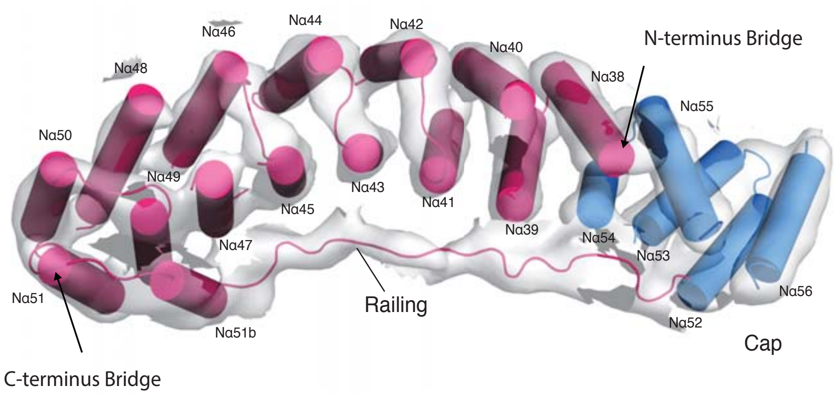

Supplementary Figure 11 | The bridge, railing and cap domains.

A close view of the bridge domain (magenta) whose C-terminus (Nα51) joins to the railing, which runs in a direction opposite to the bridge to connect to the cap domain helix Nα52 (blue). The density is shown contoured at 10σ except for the railing, which is contoured at 4σ.

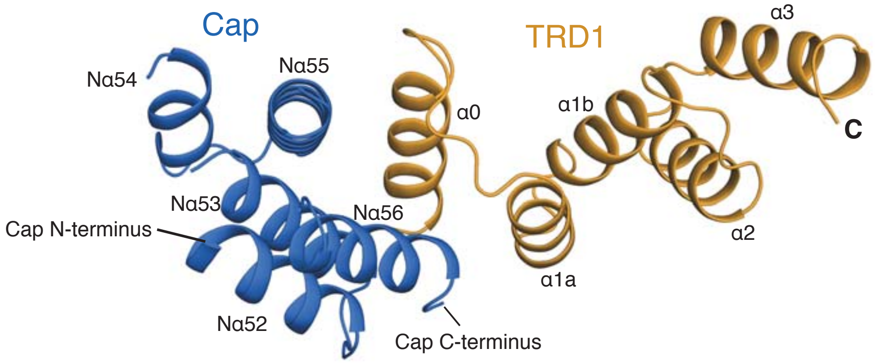

Supplementary Figure 12 | The cap/TRD1 junction.

The C-terminal helix of the cap, Nα56, leads to helix α0 at the beginning of TRD1, which was not observed in the crystal structure of the isolated FATKIN from human mTOR[9].

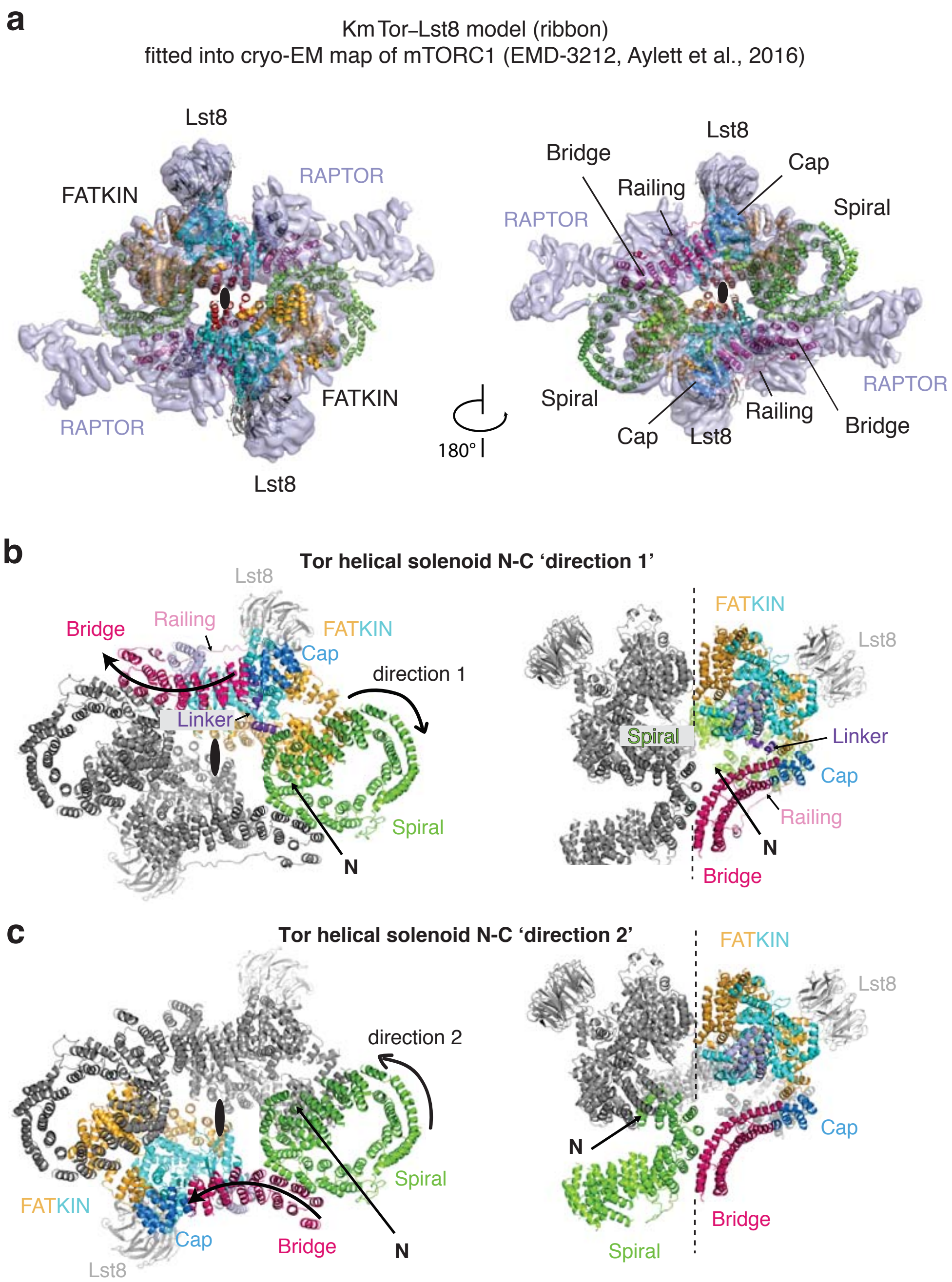

Supplementary Figure 13 | A comparison of the KmTor–Lst8 heterodimer with mTORC1.

a. The KmTor–Lst8 heterodimer superimposed on the cryo-EM density reported for mTORC1 (EMDB-3212)[10]. Two views of the complex are shown. The KmTor–Lst8 heterodimers (colored by domain) appear to be slightly tilted with respect

to the two-fold axis, however, the dimeric arrangement of KmTor–Lst8 heterodimers is nearly identical to the dimeric arrangement of human mTOR–LST8 in mTORC1.

b. RFP-verified topology ('direction 1') of one Tor monomer (colored by domains) is shown in the context of the Tor dimer. The N-terminus of the spiral (green) is free, and the C-terminus contacts the FAT domain (orange). The N-terminus of the bridge is adjacent to the cap, and the C-terminus extends away from the FATKIN. The spiral and the bridge form no intramolecular contact.

c. The alternative topology ('direction 2') for a Tor monomer (colored by domains) proposed by the mTORC1 study[10]. This topology reverses the directions of the spiral and the bridge, and consequently the spiral and the bridge form an intramolecular interaction.

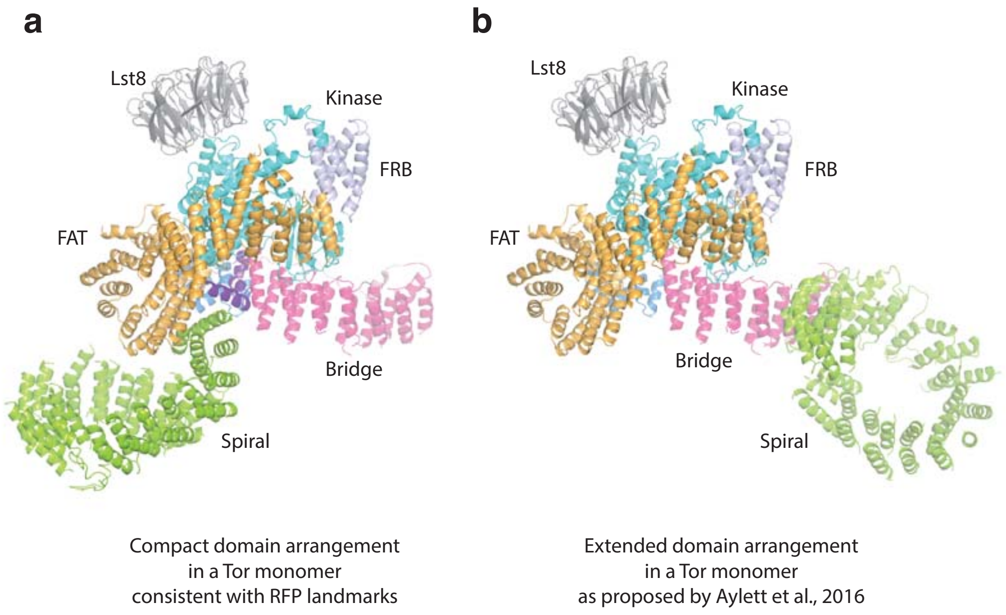

Supplementary Figure 14 | Two alternative interpretations of domain arrangements in a Tor monomer give rise to very different monomeric architectures.

a. The arrangement of domains in Tor arising from the spiral and bridge having the topology verified by the RFP insertions**.** This gives rise to a somewhat more compact arrangement of domains in which both the spiral and cap make intramolecular contacts with the FATKIN.

b. The arrangement of domains in Tor as proposed for mTORC1[10]. In addition to changing the N-to-C-terminal directions for both the spiral and bridge, this arrangement results in a somewhat more extended monomer in which only the cap but not the spiral makes an intramolecular contact with the FATKIN.

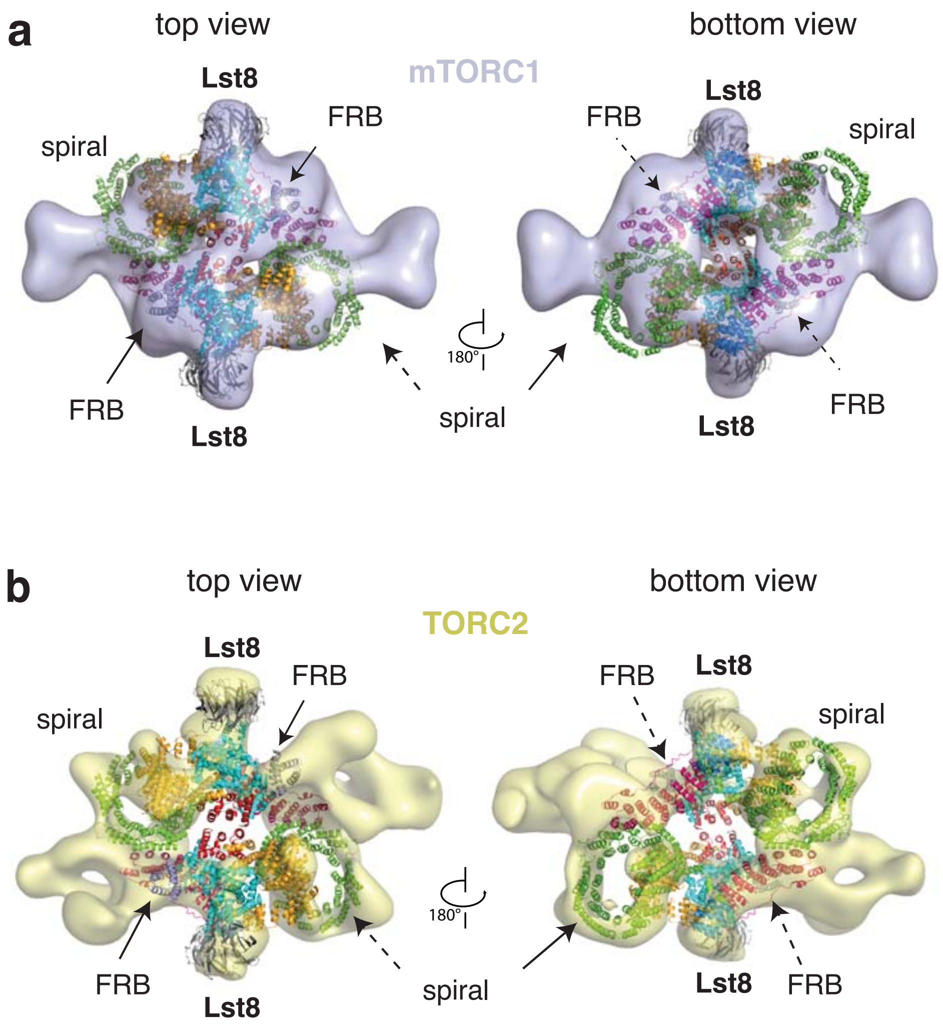

Supplementary Figure 15 | Comparison of the KmTor–Lst8 complex with the low-resolution EM-structure reported for human mTORC1 and for *S. cerevisiae* TORC2.

a. The dimer of heterodimers present in the KmTor–Lst8 was optimally fit into the density of the mTORC1 complex (EMDB-5197)[11] by Chimera. There are no overlaps and the correlation is 0.86. In this superposition, the rings of the spiral are out of density, but the rings match features present in the reference-free 2D classes of the low-resolution study[11]. The density previously ascribed to PRAS40 fits well to the Lst8.

b. Superposition of two KmTor–Lst8 heterodimers on the low-resolution EM-density for the TORC2 (EMDB-2990)[12]. Chimera was used to optimise the superposition, while allowing Lst8 to occupy the lobe of density previously identified as Avo1[12]. The entire structure is accommodated well in the density, including the spirals. The correlation is 0.78.

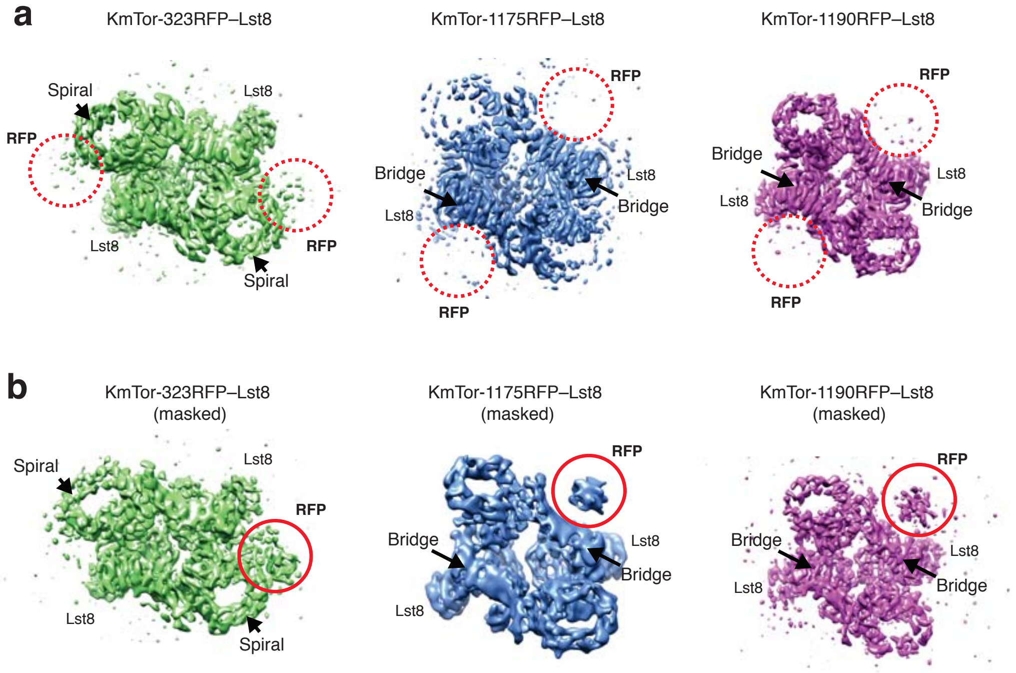

Supplementary Figure 16 | KmTor-RFP–Lst8 masked classification with density subtraction on a single tandem RFP tag.

a. The KmTor-323RFP–Lst8 (*left*), the KmTor-1175RFP (middle) and KmTor-1190RFP–Lst8 (*right*) 3D volume reconstructions at 8.5 Å, 8.7 Å and 8.5 Å resolutions, respectively, obtained after a classic data processing show weak densities for the tandem RFP tags (dashed circled red) adjacent to the spiral and the bridge, respectively.

b. KmTor-323RFP–Lst8 (*left*), KmTor-1175RFP–Lst8 (middle) and KmTor-1190RFP–Lst8 (*right*) 3D volume reconstructions at 9.1 Å, 10.3 Å and 10.5 Å resolutions, respectively, after masked classifications on a single RFP tag with signal subtraction (See Methods). Clear density for the RFP shows on the masked side of the KmTor–Lst8 cryo-EM map for the 323 tandem RFP (map on the *left*), the 1175 tandem RFP (map in the middle) and the 1190 tandem RFP (map on the *right*), and is circled red.

Supplementary Table 1. Summary of cryo-EM data collection and processing

	KmTor–Lst8 (1)	KmTor–Lst8 (2)	KmTor–Lst8 (3)	KmTor–Lst8 (4)	323 tandem RFP (5)	1175 tandem RFP (6)	1190 tandem RFP (7)
Microscope	Titan Krios	Titan Krios	Titan Krios	Titan Krios	Polara	Titan Krios	Titan Krios
Voltage (keV)	300	300	300	300	300	300	300
Detector	Falcon II	Falcon II	Falcon II	Falcon II	Falcon III	Falcon II	Falcon II
Frames	68	34	34	34	34	34	25
Exposure time (s)	4	2.5	1.5	1.5	1.5	1.5	1.5
Total dose (e-/Å)	40	40	40	40	40	40	35
Micrographs[a]	487	756	693	883	678	465	518
Pixel size (Å)	1.72	1.33	1.33	1.33	1.34	1.34	1.34
Box size	300	320	320	320	320	300	300
Raw particles[b]	37,318	42,000	52,983	102,552	49,668	59,247	36,060
Final particles	17,112		28,877		10,386	5,253	5,191
Final resolution[c]	8.5 Å		6.1 Å		9.1 Å	10.3 Å	10.5 Å

[a] number of micrographs used for processing
[b] number of particles extracted after autopicking and manual selection
[c] Gold standard FSC at 0.143 criterion

Supplementary Table 2. Summary of model building

Protein	Domain	Model type	Building and Fitting
**Tor**	SPIRAL	Backbone model with residues assigned, taking into account predicted helices	Helices and some connecting loops identified and fit into density using Coot
	LINKER	Partial backbone model with residues assigned, taking into account predicted helices	Three helices and connecting loops fit into density identified at 3σ using Coot, length of helices assigned using HHpred
	BRIDGE	Backbone model with residues assigned, taking into account predicted helices	Helices and some connecting loops identified and fit into density using Coot
	RAILING	Backbone model with residues assigned, taking into account predicted helices	One helix identified and fit into density using Coot, long tubular density identified at 3σ fit with a long linker assigned using the HHpred
	CAP	Backbone model with residues assigned, taking into account predicted helices	Five helices identified and fit into density using Coot
	FATKIN	Backbone model with residues assigned	KmTor FATKIN homology model generated by SWISS-MODEL using mTOR FATKIN (PDB ID 4JSV) as initial model, then fit into density and manually adjusted using Coot
**Lst8**	WD40	Backbone model with residues assigned	KmLst8 homology model generated by SWISS-MODEL using mLST8 (PDB ID 4JSV) initial model and then fit into density using Coot

Supplementary references

1. Rosenthal, P. B. & Henderson, R. Optimal determination of particle orientation, absolute hand, and contrast loss in single-particle electron cryomicroscopy. *J Mol Biol* **333,** 721–745 (2003).
2. Kucukelbir, A., Sigworth, F. J. & Tagare, H. D. Quantifying the local resolution of cryo-EM density maps. *Nat Meth* **11,** 63–65 (2014).
3. Scheres, S. H. W. RELION: implementation of a Bayesian approach to cryo-EM structure determination. *J. Struct. Biol.* **180,** 519–530 (2012).
4. Pettersen, E. F. *et al.* UCSF Chimera--a visualization system for exploratory research and analysis. *J Comput Chem* **25,** 1605–1612 (2004).
5. Edgar, R. C. MUSCLE: multiple sequence alignment with high accuracy and high throughput. *Nucleic Acids Res.* **32,** 1792–1797 (2004).
6. Waterhouse, A. M., Procter, J. B., Martin, D. M. A., Clamp, M. & Barton, G. J. Jalview Version 2--a multiple sequence alignment editor and analysis workbench. *Bioinformatics* **25,** 1189–1191 (2009).
7. Robert, X. & Gouet, P. Deciphering key features in protein structures with the new ENDscript server. *Nucleic Acids Res.* **42,** W320–W324 (2014).
8. Wall, M. A., Socolich, M. & Ranganathan, R. The structural basis for red fluorescence in the tetrameric GFP homolog DsRed. *Nat. Struct. Biol.* **7,** 1133–1138 (2000).
9. Yang, H. *et al.* mTOR kinase structure, mechanism and regulation. *Nature* **497,** 217–223 (2013).
10. Aylett, C. H. S. *et al.* Architecture of human mTOR complex 1. *Science* **351,** 48–52 (2015).
11. Yip, C. K., Murata, K., Walz, T., Sabatini, D. M. & Kang, S. A. Structure of the human mTOR complex I and its implications for rapamycin inhibition. *Mol. Cell* **38,** 768–774 (2010).
12. Gaubitz, C. *et al.* Molecular Basis of the Rapamycin Insensitivity of Target Of Rapamycin Complex 2. *Mol. Cell* **58,** 977–988 (2015).
